# A Review on Multifunctional Polymer–MXene Hybrid Materials for Electronic Applications

**DOI:** 10.3390/molecules30091955

**Published:** 2025-04-28

**Authors:** Fatemeh Morshedi Dehaghi, Mohammad Aberoumand, Uttandaraman Sundararaj

**Affiliations:** Department of Chemical and Petroleum Engineering, University of Calgary, Calgary, AB T2L1Y6, Canada

**Keywords:** MXene, polymer nanocomposites, electrical conductivity, dielectric properties, electromagnetic interference shielding, multifunctionality

## Abstract

MXenes, a family of two-dimensional (2D) transition metal carbides, carbonitrides, and nitrides, have emerged as a promising class of nanomaterials for interdisciplinary applications due to their unique physiochemical properties. The large surface area, excellent electrical conductivity, superior mechanical properties, and abundant possible functional groups make this layered nanomaterial an ideal candidate for multifunctional hybrid materials for electronic applications. This review highlights recent progress in MXene-based hybrid materials, focusing on their electrical, dielectric, and electromagnetic interference (EMI) shielding properties, with an emphasis on the development of multifunctionality required for advanced electronic devices. The review explores the multifunctional nature of MXene-based polymer nanocomposites and hybrid materials, covering the coexistence of a diverse range of properties, including sensory capabilities, electromagnetic interference shielding, energy storage, and the Joule heating phenomenon. Finally, the future outlook and key challenges are summarized, offering insights to guide future research aimed at improving the performance and functionality of MXene–polymer nanocomposites.

## 1. Introduction

Over the past few decades, rapid industrial and technological advancements have driven an unprecedented demand for high-performance, lightweight electronic devices [1,2,3]. The continued miniaturization and integration of electronic devices are fueled by increasing energy consumption and associated environmental concerns. These factors have imposed stringent demands on materials used in advanced technologies, such as wearable electronics and soft robotics [4,5,6,7,8,9]. These materials must not only be lightweight and flexible but must also be multifunctional [10,11]. In fact, modern electronic hardware materials have evolved beyond serving a singular purpose. Instead, they are expected to perform multiple functions simultaneously without adding significant weight, bulk, or cost [4,8,10]. Multifunctionality enables components to address challenges such as energy storage, electromagnetic interference (EMI) shielding, sensing, and thermal management within a single, integrated system [12,13,14,15]. Among various strategies, the integration of nanomaterials into polymer matrices, along with precise microstructure design, has emerged as a promising approach [16,17,18,19,20]. The choice of polymers and nanomaterials—spanning from zero-dimensional (0D), one-dimensional (1D), and two-dimensional (2D) to the three-dimensional (3D)—depends largely on the target applications [18,19,20].

**Figure 1:**
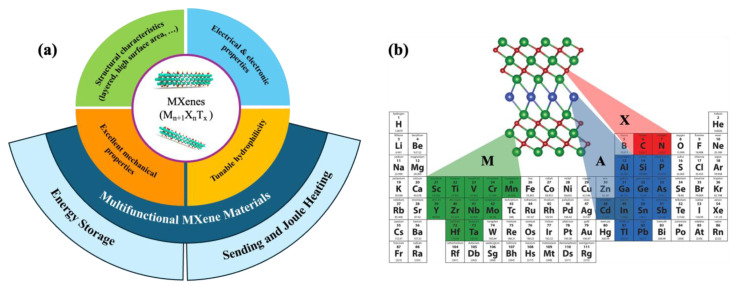
(**a**) Schematic illustration of multifunctional polymer–MXene nanocomposites in electronics. (**b**) Forming elements of M_n+1_AX_n_ MAX phase based on the periodic table [83]. Copyright 2019, Ceramics International.

Since the discovery of graphene, 2D nanomaterials have garnered significant attention due to their large surface area, excellent properties, and suitability for electronic applications [21,22,23]. Polymer nanocomposites, in particular, combine the lightweight nature, flexibility, and ease of processing of polymers with the unique layered structure and high surface area of 2D nanomaterials [23,24,25,26]. Like graphene, transition metal carbides and/or nitrides, known as MXenes, have emerged as next-generation materials for flexible electronics due to their exceptional properties. These properties include high electrical conductivity, mechanical strength, layered structure, and tunable surface functional groups, as highlighted in Figure 1a [3,27,28,29]. The polymer–MXene composites have been particularly recognized as an innovative class of materials, leveraging the exceptional properties of MXenes alongside the versatility and processability of polymers to enable a wide range of high-performance applications [2,30]. This synergy paves the way for advanced opportunities in the study and development of multifunctional material systems. Therefore, it offers the potential for next-generation electronic devices that are more flexible, lightweight, and exhibit even better performance [31]. Devices such as energy storage, sensing, and Joule heating systems are essential for advancing smart technologies [14,32,33].

For energy storage applications, MXenes have been utilized in batteries (as electrodes and electrolytes), supercapacitors, and dielectric materials for capacitors [34,35,36,37,38]. In electrode materials, the exceptional electrical conductivity and tunable interlayer spacing of MXenes, compared to transition metal dichalcogenides (TMDCs), make them ideal candidates for this application [39,40]. These advantages become even more significant when MXenes are combined with conducive polymers [41,42]. Therefore, the incorporation of MXenes enhances multiple functionalities, such as capacitance, coulombic efficiency, and cycle stability, while also providing additional benefits like anti-corrosion properties and actuator performance for smart devices [42,43,44]. In electrolytes, MXene–polymer nanocomposites, especially gel polymer electrolytes, have shown significant potential for all-solid-state batteries by improving ionic conductivity and effectively suppressing dendrite formation [35,45]. In supercapacitors, MXene electrodes combined with conductive polymers deliver high capacitance, excellent cycling stability, and improved charge/discharge rates [46]. One of the key advantages of MXenes over graphene is their hydrophilic nature, which enhances the wettability of electrodes with electrolytes, thereby improving ion transport [47]. For capacitors, the integration of MXenes into polymer matrices enables a high dielectric constant. The presence of surface functional groups facilitates stronger interfacial polarization compared to TMDC and graphene [48]. Additionally, tuning the interlayer spacing and surface functionalization through optimized synthesis and processing techniques can minimize dielectric loss and enhance breakdown strength [49]. For instance, MXene–poly(vinylidene fluoride) (PVDF) composites exhibit a dielectric constant 25 times higher than pure PVDF due to the alignment of MXene nanosheets and interfacial polarization [50,51].

Wearable electronics, such as smart textiles, health monitors, and flexible displays, require materials that integrate mechanical flexibility and durability, high sensitivity to external stimuli (strain, temperature, and/or humidity), effective thermal management, and Joule heating capabilities. MXene–polymer sensors have demonstrated exceptional performance to detect human body motion with high sensitivity across a sensing range of 0–100% strain [52]. Furthermore, MXene-coated fabrics exhibit effective Joule heating performance for adaptive thermal regulation, which is critical for wearable thermal management systems [53]. Soft robotics and artificial skins require materials that mimic the properties of biological tissues, including stretchability, self-healing, and sensory responsiveness [54,55,56,57]. These materials hold significant potential for advancing prosthetics, human–machine interfaces, and soft robotic components [58,59]. Compared to other 2D materials, like graphene, that have been used for this purpose, MXene materials have shown an edge. It is mainly due to its exceptional electrical conductivity of 2.4 × 10^4^ S/cm [49], which is three orders of magnitude higher than that of graphene (106 S/cm) [60], as well as its inherent hydrophilicity, which eliminates the need for challenging surface functionalization processes that could introduce defects and reduce conductivity [61]. It is worth mentioning that the metallic kind of conductivity and the tunable surface chemistry of MXenes bring an exceptional temperature sensitivity compared to other well-studied 2D materials like graphene and TMDCs [61,62]. Also, MXene materials have shown a competitive performance for humidity sensing compared to GO, thanks to maintaining high electrical conductivity while possessing abundant hydrophilic terminal groups, fast response time, and stability [63,64]. With the rise of wireless communication and compact electronics, EMI shielding is crucial to prevent electromagnetic pollution and ensure reliable device performance [13,65]. MXene–polymer composites uniquely combine electrical conductivity, which is needed for reflection, and dielectric polarization for promoting absorption [66]. MXene’s layered structure and tunable surface chemistry enable efficient conductive networks, enhancing shielding efficiency without sacrificing flexibility [67,68]. Compared to other 2D materials, like graphene, the combination of a layered structure with the metallic electrical conductivity and polar surface groups of MXenes has made it stand out for EMI shielding with high absorption [69,70]. It can be comprehended by comparing the EMI-shielding effectiveness of 92 dB (45 μm sheet) and 35 dB (thickness N/A), corresponding to MXene and graphene sheets, respectively [71,72,73].

Despite MXene material’s impressive multifunctional properties, several challenges must be addressed to enable its commercialization. These include environmental concerns related to the use of fluorine-containing chemicals during synthesis, susceptibility to oxidation leading to performance degradation, poor interfacial bonding between intrinsically hydrophilic MXene and nonpolar polymers, and the difficulty of balancing multiple desired properties [74,75,76,77,78,79,80]. This review highlights the critical role of multifunctional polymer–MXene hybrid materials in addressing the challenges of fast-progressing modern electronics. These materials offer unique combinations of electrical, dielectric, electromagnetic, sensing, thermal, and mechanical properties, enabling applications in energy storage, wearable electronics, soft robotics, EMI shielding, and thermal management systems. The review begins with an overview of common synthesis methods for MXene production and their fundamental properties that are relevant to electronic applications, including electrical, dielectric, and mechanical characteristics. It then explores the role of MXenes in enhancing these properties, focusing on two key areas: energy storage and sensing/Joule heating applications. This review concludes with insights into future research directions and the remaining challenges in this field.

### 1.1. MXene Structure

In recent decades, 2D nanomaterials have attracted considerable interest due to their excellent electrical and electronic properties [46]. Among these materials, transition metal carbides and/or nitrides, known as MXenes, have stood out as particularly promising since their discovery in 2011 at Drexel University [31]. MXenes are characterized by the general formula M_n+1_X_n_T_x_, where M represents a transition metal, X is carbon and/or nitrogen, and T denotes surface functional groups, such as -O, -OH, -F, and -Cl [31,81]. Typically, the layered structure of MXenes is synthesized from their bulk parent materials, MAX phases, through selective chemical etching of the A layer, which consists of elements from group 13 or 14 elements [82]. Figure 1b illustrates the chemical structure of the MAX phase, represented by the chemical formula M_n+1_AX_n_ [83].

The outstanding characteristics of these advanced 2D nanomaterials, including their large surface area, high metallic conductivity, tunable hydrophilicity, and excellent electronic performance, make them ideal candidates for multifunctional electronic applications. These applications include energy storage devices, EMI shielding, and sensors, particularly when integrated into polymer matrices [33,46,52,84].

### 1.2. MXene Synthesis

Since the discovery of MXene, and considering their exceptional properties, significant research attention has focused on developing various types of MXenes and optimizing their synthesis approaches [31,85,86]. This section outlines the most common MXene synthesis methods, including direct and indirect (using fluoride salts), hydrothermal, and electrochemical etching.

#### 1.2.1. Direct and Indirect (MILD) Synthesis Process

**Figure 2:**
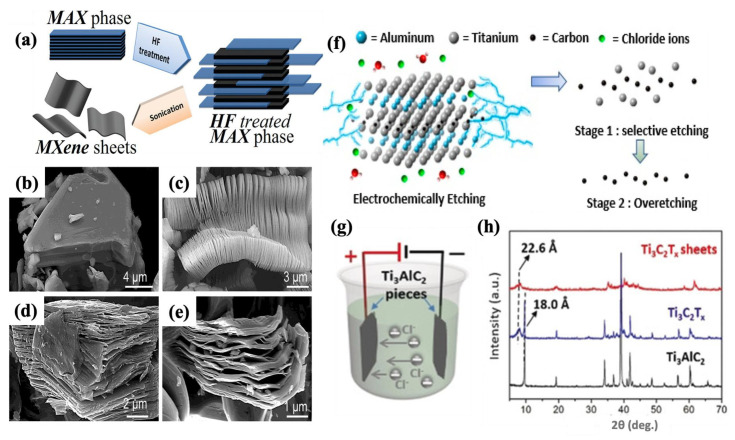
(**a**) Schematic illustrating the etching and exfoliation process of MAX phase [87]. SEM images of (**b**) Ti_3_AlC_2_ before HF treatment, (**c**) Ti_3_AlC_2_ after HF treatment, (**d**) Ti_2_AlC after HF treatment, and (**e**) Ta_4_AlC_3_ after HF treatment [87]. Copyright 2012, American Chemical Society. (**f**) Electrochemical etching synthesis method of MAX phase in a HCl electrolyte solution [80]. Copyright 2019, American Chemical Society. (**g**) Electrochemical cell setup for the electrochemical etching synthesis method, and (**h**) XRD patterns of Ti_3_AlC_2_ and Ti_3_C_2_T_x_ MXene before and after intercalation step [94]. Copyright 2019, Wiley-VCH.

The direct etching of MXenes from the MAX phase using powerful chemical etchants, like hydrofluoric acid (HF), is the most straightforward and widely used synthesis approach to date. This process, first performed by Naguib et al., led to the discovery of MXenes [31]. As illustrated in Figure 2a, they used a 50% HF solution to etch Ti_3_AlC_2_ for 2 h at room temperature, successfully removing the Al layers. This efficient and fast process yields high-quality MXene with a complete removal of the A layers, producing multilayered MXene with closely packed Ti-C interlayers. To expand the interlayer spacing and produce delaminated Ti_3_C_2_T_x_, chemical methods (e.g., using solvents such as isopropyl amine, water, dimethyl sulfoxide (DMSO), and hydrazine), followed by mechanical methods (e.g., sonication), were applied [86,87,88]. This technique has also been successfully applied to other MAX phases, including Ti_2_AlC, Ta_4_AlC_3_, (Ti_0.5_Nb_0.5_)_2_AlC, (V_0.5_Cr_0.5_)_3_AlC_2_, and Ti_3_AlC_2_ [28,87,89]. Furthermore, numerous studies have investigated the effects of etching parameters, such as the HF concentration, time, and temperature, to optimize conditions for producing highly efficient and homogeneous MXenes [90]. Figure 2b–e shows SEM images of Ti_3_AlC_2_ before HF treatment and different types of MXenes after HF treatment, clearly confirming successful exfoliation.

Given the corrosive nature and extreme hazards of HF, indirect-etching methods have been introduced as safer and more environmentally friendly alternatives, though they are less efficient and much slower [49]. This gentler method uses a combination of acids (e.g., hydrochloric acid (HCl) or sulfuric acid (H_2_SO_4_)) and fluorine-containing salts (e.g., lithium fluoride (LiF), sodium fluoride (NaF), and potassium fluoride (KF)) [91]. In addition to removing the A layer from the MAX phase, the interaction of the formed cations with water molecules increased interlayer spacing, enhanced stability, and reduced defects. Effective parameters in this method include etching time and etchant concentration. Mo_2_CT_x_, Ti_3_CNT_x_, Ti_3_C_2_T_x_, and (Nb_0_._8_Zr_0.2_)_4_C_3_T are among the MXenes synthesized using this method [28,49,89].

#### 1.2.2. Electrochemical Etching

The synthesis of MXene can be performed using an electrochemical etching method, which removes A layers from the MAX phase by applying an electric field [92]. In this process, electrons are transferred from the A layer in an electrolyte solution, selectively etching the A layer of the MAX phase. An appropriate design of electrolyte is necessary for achieving a selective etching of MAX phase with a commonly used HF solution or fluorine-free electrolyte solution, such as HCl and sodium chloride (NaCl). Performing the electrochemical etching process of the Ti_3_AlC_2_, Ti_2_AlC, and Ti_3_SiC_2_ MAX phases can result in the removal of both A (Al/Si) and Ti atoms in an over-etching stage, leading to amorphous carbon layer formation (as shown in Figure 2f) [80]. For instance, Sun et al. [93] used a low concentration of HCl solution as an electrolyte to remove the A layer. However, the subsequent over-etching process reduced the final efficiency and prolonged the process by hindering carbide-derived carbon layer (CDC) formation.

To address this challenge, researchers have focused on selective A layer removal. In a study performed by Yang et al. [94], Ti_3_C_2_T_x_ MXene was successfully synthesized using anodic corrosion in a binary aqueous electrolyte system containing 1 M of ammonium chloride (NH_4_Cl) and 0.2 M of tetramethylammonium hydroxide (TMAOH). In this process, Cl ions from the electrolyte penetrate the MAX layers on the anode electrode, etching the Al layer, while ammonium hydroxide ions (NH_4_OH) expand interlayer spacing via an intercalation effect. Figure 2g illustrates the electrochemical cell configuration, and Figure 2h shows XRD results confirming successful MXene synthesis. This method expanded the capability of these MXene materials in supercapacitors by providing a high yield of over 90% and introduced an environmentally friendly approach based on a fluorine-free synthesis procedure. Moreover, this method has been extended to synthesize other types of MXene, including Ti_2_CT_x_, Cr_2_CT_x_, and V_2_CT_x_ using various electrolyte and electrochemical cell setups [95,96].

#### 1.2.3. Hydrothermal Process

**Figure 3:**
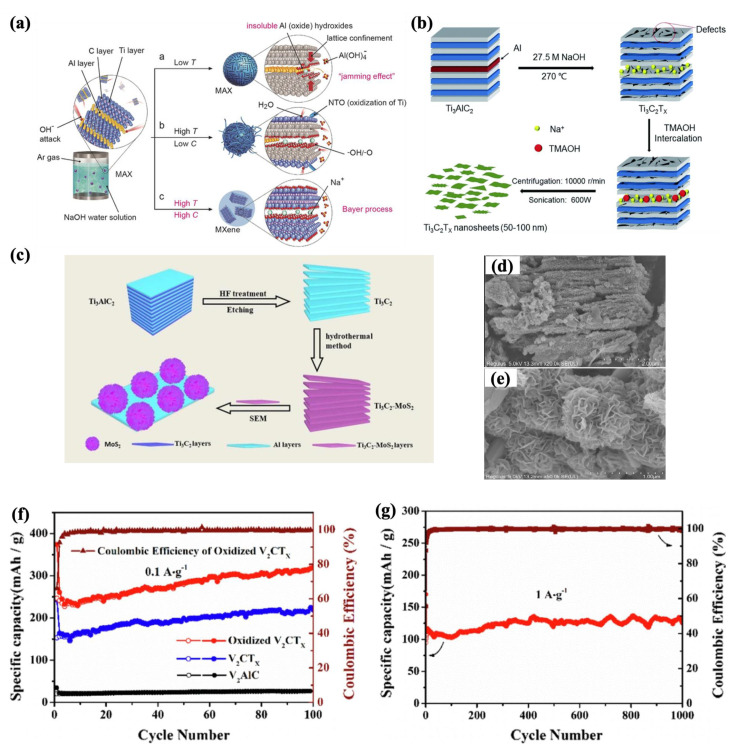
(**a**) Hydrothermal MXene synthesis process in NaOH solution under different processing conditions, which led to different morphology [102]. Copyright 2019, Wiley-VCH. (**b**) Synthesis of delaminated MXene nanosheets using hydrothermal method, followed by TMAOH intercalation and sonication process [103]. Copyright 2019, Royal Society of Chemistry. (**c**) Schematic illustrating Ti_3_C_2_-MoS_2_ composite by applying a combination of HF treatment, hydrothermal, and freeze-drying processes [108]. (**d**,**e**) SEM images of Ti_3_C_2_-MoS_2_ composite with scale bar 2 μm and 1 μm, respectively, showing the flower-like structure [108]. Copyright 2022, Elsevier. (**f**,**g**) Cycling performance of V_2_CT_x_ MXene at 100 mA·g^−1^ and 1000 mA·g^−1^ after 100 and 1000 cycles, respectively [109]. Copyright 2020, Elsevier.

To address the environmental and health hazards of HF, hydrothermal methods have emerged as fluorine-free alternatives [97,98]. In this method, the MAX phase precursor is exposed to high temperatures and pressures for a specific duration [99]. This method yields high-quality MXene with reduced defects and tailored morphologies while enabling desired surface functionalization in a single step. However, optimizing processing conditions, such as temperature, time, and pressure, remains a key challenge [99,100,101]. Li et al. [102] synthesized multilayered MXene (Ti_3_C_2_T_x_) with 92% purity using a 27.5 M sodium hydroxide (NaOH) solution at 270 °C under an argon atmosphere (as shown in Figure 3a). Optimizing the NaOH concentration and temperature prevented the formation of impurity compounds of Na_2_Ti_3_O_7_/Na_2_Ti_5_O_11_ and insoluble products of Al(OH)_3_/AlO(OH), achieving high yields. In a subsequent study, the same group used TMAOH and high-power ultrasonication (i.e., 600 W) to delaminate these multilayered nanosheets into few-layered MXenes (Figure 3b) [103].

It is worth mentioning that many studies combine hydrothermal methods with other techniques, such as HF etching, to create hybrid structures or expedite the process [104,105,106,107,108]. For instance, Guo et al. [108] conducted a hydrothermal process (Figure 3c) on Ti_3_C_2_T_x_ MXene, synthesized by the HF etching method and combined with MoS_2_, to produce a flower-like composite (Figure 3d,e). This composite exhibited a 60% increase in surface area compared to the sample without MoS_2_. Similarly, Luo et al. [109] synthesized an oxidized V_2_CT_x_ MXene enriched with functional VO_2_ and O groups using hydrothermal processing in an H_2_O_2_ environment. As shown in Figure 3f,g, this method provided an improved capacitance of 318 mAh·g^−1^ at 100 mA·g^−1^ after 100 cycles, and 125 mAh·g^−1^ at 1000 mA·g^−1^ after 1000 cycles, along with better cycling stability. This method has potential for anode materials in lithium-ion battery applications.

#### 1.2.4. Quality, Stability, and Scalability of MXene Synthesis: Industrial Considerations

The large-scale production of MXenes for industrial applications depends on two crucial factors: (1) application-based properties, including the quality and stability of synthesized MXene, and (2) scalability, which involves feasibility, cost, and environmental considerations. From an application standpoint, the quality and stability of MXene are directly influenced by the synthesis method. In the direct-etching method with HF, while the concentration of HF etchant can be adjusted, the presence of –F termination groups can negatively affect MXene stability and limit its final applications. Indirect-etching methods (e.g., using LiF and HCl) offer an alternative, with improved MXene quality and stability, though at the expense of a slower process [28]. Electrochemical etching and hydrothermal synthesis provide further control over surface terminations and enhance MXene quality, making them more suitable for application-specific modifications [110,111].

When considering scalability, multiple factors must be evaluated, including safety, environmental impact, production cost, and large-scale feasibility. Despite being the most commonly used technique, HF direct etching presents significant challenges due to the hazardous nature of HF, requiring strict handling procedures and costly waste disposal, which increase overall production costs. However, the simultaneous etching and intercalation process in this method is advantageous, potentially reducing synthesis time and cost. The MILD synthesis method, while safer and more environmentally friendly, suffers from lower efficiency and a slower production rate, impacting its cost-effectiveness for large-scale production. In contrast, electrochemical etching, a fluorine-free method, eliminates HF-related safety concerns. However, scaling up electrochemical cells for large-volume production is cost-intensive due to the complexity of setting up large electrode systems. Similarly, although hydrothermal synthesis offers a fluorine-free approach with tailored surface chemistry, it is limited by harsh processing conditions, such as high temperatures and pressures [112,113,114].

Table 1 provides a summary of the key advantages and challenges of each synthesis method in terms of application-based properties and scalability.

## 2. MXene Functionalities for Electronic Applications

### 2.1. Electrical Property

The presence of metallic groups within the MXene structure is the primary factor contributing to its high electrical conductivity [83,115]. However, additional parameters, including the type of MAX phase, synthesis methods, and post-synthesis treatments, significantly influence the final conductivity values. For Ti_3_C_2_T_x_ MXene, these values can range from 1 S.cm^−1^ to as high as 24,000 S.cm^−1^ [116]. These parameters impact crucial factors such as defect concentration, the nature and proportion of surface terminations (-F, -OH, =O, and -Cl), delamination efficiency, interlayer spacing (d-spacing) of MXene sheets, and the lateral size of the synthesized material [117]. Table 2 summarizes the influence of various synthesis conditions on the corresponding electrical conductivity values.

#### Theoretical Background and Manipulation of Electrical Conductivity in MXene–Polymer Nanocomposites

Considering the growing application of electrically conductive polymer nanocomposites in electronic devices with various functionalities, it is essential to understand the fundamentals of electrical conductivity and its critical parameter, the electrical percolation threshold [126,127]. MXene nanomaterials, along with other conductive nanofillers such as carbon-based nanomaterials, have emerged as outstanding candidates for enhancing electrical conductivity within polymer matrices [128,129]. The percolation threshold represents the critical filler concentration at which an insulating polymer transitions into a conductive composite material [130]. As the nanofiller concentration increases, conductive pathways are formed, enabling electron transport through the polymer matrix by connecting or contacting neighboring nanofillers. According to percolation theory, the electrical conductivity (*σ*) of a polymer nanocomposite depends on the filler volume fraction (*v*) and can be expressed by the following equation:

(1) $$\sigma =\sigma_{0}{\left(\nu -\nu_{c}\right)}^{t}$$

where σ_0_ is the intrinsic conductivity of the filler, *v*_*c*_ represents the electrical percolation threshold, and t is the critical power-law exponent. This theory explains the sharp transition from insulating to conductive behavior in polymer nanocomposites as the filler concentration increases. Understanding this behavior plays an important role in optimizing the performance of these materials across various applications. Moreover, numerous studies have focused on reducing the nanofiller content required to reach the percolation threshold, in order to lower production costs while maintaining optimal functionality [131].

**Figure 4:**
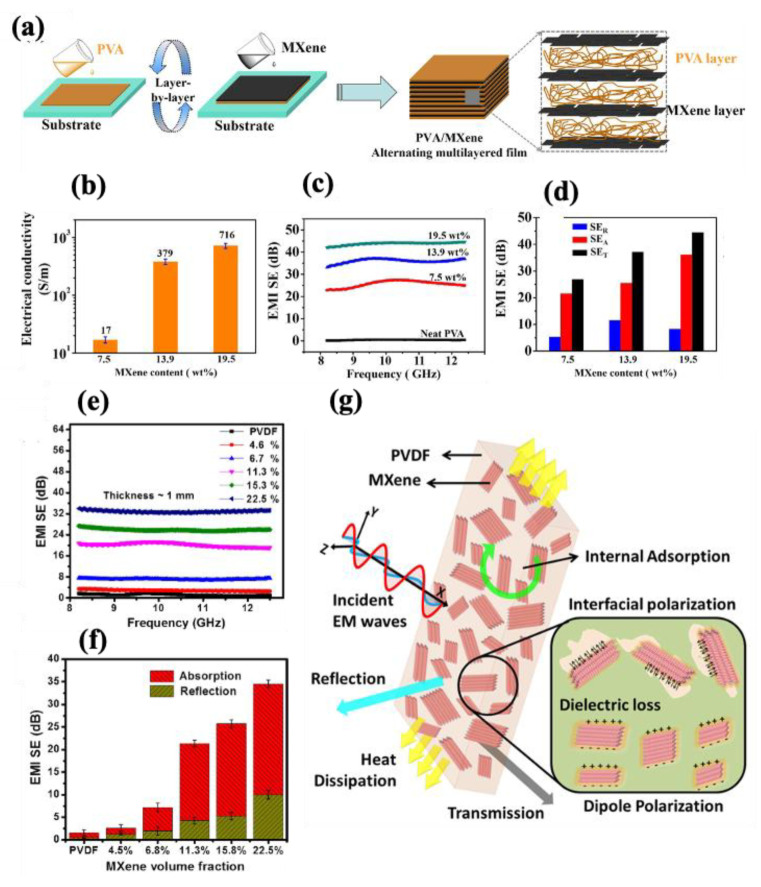
(**a**) Schematic illustrating preparation method of PVA–MXene alternating multilayered nanocomposite films, (**b**) electrical conductivity, (**c**) EMI SE, and (**d**) SE_A_, SE_R_, and SE_T_ of PVA–MXene multilayered nanocomposite films with different MXene contents [132]. Copyright 2019, Elsevier. (**e**) EMI SET and (**f**) SE_A_, and SE_R_ of PVDF–MXene nanocomposites as a function of frequency with varying MXene contents (1 mm thickness), and (**g**) schematic illustrating EMI-shielding mechanism for PVDF/MXene nanocomposite film [134]. Copyright 2019, Elsevier.

To meet the requirements of a wide range of electronic applications, many studies have explored the incorporation of MXene nanomaterials into specific polymers using various processing techniques to achieve tailored properties. Jin et al. [132] fabricated polyvinyl alcohol (PVA)–MXene nanocomposite multilayered films, achieving significant enhancements in EMI shielding and thermal conductivity. Their alternative multilayered structure (illustrated in Figure 4a), containing 19.5 wt% MXene, demonstrated electrical conductivity of 716 S·m^−1^ (Figure 4b) and an EMI-shielding effectiveness (EMI SE) of 44.4 dB, primarily through an absorption mechanism (Figure 4c,d). Additionally, the films exhibited enhanced in-plane thermal conductivity (4.57 W·m^−1^·K^−1^) and anti-dripping performance, reinforcing their potential for advanced electronic devices. Feng et al. [133] developed conductive epoxy (EP)–MXene nanocomposites by solution blending, highlighting their applicability as electrically conductive adhesives. With the addition of 1.2 wt% of MXene into the epoxy matrix, the d-spacing increased between the MXene nanosheets, facilitating a rise in electrical conductivity to 4.52 × 10^−4^ S·cm^−1^. In another study focused on EMI shielding and multifunctional electronics, Rajavel et al. [134] produced cost-effective PVDF–MXene nanocomposites using solution mixing followed by compression molding. Nanocomposites with a 22.55 vol% MXene achieved an electrical conductivity of 0.988 S.m^−1^, a thermal conductivity of 0.767 W.m^−1^ K^−1^, and an EMI SE of 34.49 dB (at a thickness of 1 mm), with absorption as the dominant shielding mechanism (Figure 4e,f). These properties were attributed to the formation of conductive networks and micro-capacitors within the polymer matrix. As shown in Figure 4g, enhanced interfacial polarization improved the dielectric properties, resulting in a high dielectric constant and low dielectric loss, which effectively attenuated electromagnetic waves. Table 3 summarizes various multifunctional MXene–polymer nanocomposites, highlighting their enhanced electrical properties and diverse applications in advanced electronics.

### 2.2. Dielectric Properties

MXene’s partially metallic nature enhances electrical conductivity and polarizability, which gives superior dielectric properties. The presence of surface terminations such as -F, -OH, and =O increases the dipolar contribution, further improving dielectric performance. The layered structure of MXene also plays a significant role in enhancing the dielectric constant through interfacial polarization [29,141]. Key factors such as flake size, interlayer spacing, and defects in MXene influence its dielectric properties. Moreover, processing conditions, including etching time, temperature, and post-processing treatments, can significantly affect these properties [142,143,144]. Xu et al. [142] investigated the impact of solvents on the dielectric properties of MXene for EMI-shielding applications. Solvents such as dimethylformamide (DMF) (f-Ti_3_C_2_T_x_), ethanol (e-Ti_3_C_2_T_x_), and DMSO (s-Ti_3_C_2_T_x_) were used to study their effects. Based on the results, the dielectric loss for s-Ti_3_C_2_T_x_ MXene was higher compared to e-Ti_3_C_2_T_x_ and f-Ti_3_C_2_T_x_, demonstrating the superior ability of s-Ti_3_C_2_T_x_ to absorb EM waves [142].

#### 2.2.1. Theoretical Background

##### Dielectric Constant and Dielectric Loss

Dielectric materials interact with electric fields due to the presence of charge carriers that can be displaced. When subjected to an electric field, these materials have the capacity to store electrical energy through charge separation, a process known as polarization. However, during each charging–discharging cycle, a portion of the energy is inevitably dissipated as heat or other forms of energy loss. The ability of a material to store an electric field is quantified by its dielectric permittivity ($\varepsilon_{r}$), which can be determined through impedance measurements [145]. As described in Equation (2), dielectric permittivity consists of two frequency-dependent components in a complex function: the real part ($\varepsilon^{\prime }$), representing the dielectric constant or energy storage capability, and the imaginary part ($\varepsilon^{\prime \prime }$), which corresponds to dielectric loss [48].

(2) $$\varepsilon_{r}=\varepsilon^{\prime }\left(\omega \right)-i.\varepsilon^{\prime \prime }\left(\omega \right)$$

The dielectric loss can also be expressed as a relative loss, known as the loss tangent (tan⁡δ), which is the ratio of $\varepsilon^{\prime \prime }$ to $\varepsilon^{\prime }$.

##### Polarization Mechanisms in Dielectric Materials

**Figure 5:**
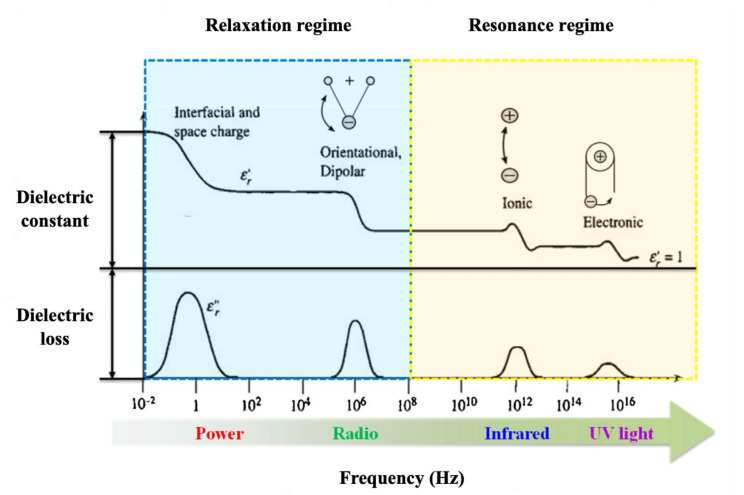
Various types of polarization mechanisms and their frequency dependences [148]. Copyright 2019, Elsevier.

The performance of dielectric materials is heavily influenced by their polarization mechanisms. When an electric field is applied, polarization refers to the total dipole moments per unit volume and is directly proportional to the dielectric permittivity through the following equation [146]:

(3) $$P=\left(\varepsilon_{r}-1\right){\;\varepsilon }_{0}E$$

where P presents the dielectric polarization,${\;\varepsilon }_{0}$ is the permittivity of a vacuum, and E is the applied electric field. Polarization mechanisms can be classified into four different types based on the relaxation and resonance regimes (Figure 5): electronic (P_e_), ionic (P_i_), dipolar or orientational (P_d_), and interfacial (P_int_) polarizations [147].

Electronic polarization (P_e_) arises from the displacement of electron clouds relative to the nucleus in the neutral atoms under the influence of an external electric field. The displacement creates an induced dipole moment, which vanishes when the electric field is removed. Electronic polarization is common in all solid dielectrics and is particularly significant at higher frequencies, such as those in the optical range, due to the rapid response of electron clouds to changes in the external electric field [146].

Ionic polarization (P_i_) occurs when an external electric field is applied to ionic crystals, and a net dipole moment is generated by the relative displacement of positive and negative ions towards the +x-axis and −x-axis directions, respectively. Similar to electronic polarization, ionic polarization disappears once the electric field is removed. It is largely independent of temperature due to the intrinsic nature of the mechanisms involved [148].

Dipolar polarization in polymers arises from the presence of polar groups and the structure of polymer chains. Applying an external electric field can align dipoles with the electric field direction. Temperature changes significantly affect the development of dipolar polarization. At extremely low temperatures, strong intermolecular interactions hinder dipole alignment, while at very high temperatures, intense thermal motion disrupts alignment [147,148].

Interfacial polarization (P_int_) occurs at the interface of heterogeneous systems, where there is an accumulation of opposite charges on each side of the interface. This phenomenon arises due to differences in conductivity and polarity components. This mechanism is particularly important in polymer nanocomposites and typically occurs in the low-frequency range, as dipoles require longer relaxation time to return to their original orientation [146].

##### Importance of EMI-Shielding Properties

Rapid advancements in technology, especially in the field of electronic devices, have created a new form of pollution, namely EMI smog [149]. This interference can be generated from various sources, including natural phenomena like lightning, as well as human-made sources such as electronic devices, wireless communication, and industrial equipment [150,151]. This energy can have a destructive effect on the normal operation of electronic devices, leading to data loss, malfunction, or complete system failure. Moreover, prolonged exposure to EM radiation can increase the risk of serious health issues in humans and animals, such as cancer, asthma, heart disease, and genetic damage [2,152,153].

**Figure 6:**
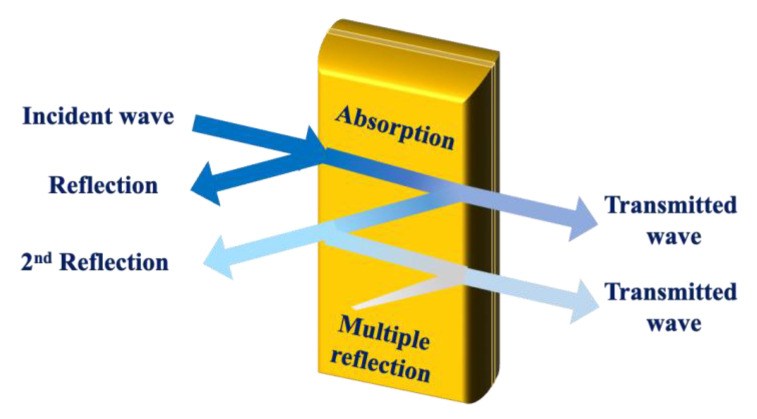
Different mechanisms for EMI shielding.

Continuous advancements in modern technology underscore the critical need for effective EMI-shielding solutions to ensure the integrity and performance of electronic systems. The primary purpose of the EMI-shielding materials is to protect electronic and electrical devices from external EMI waves and prevent internal emissions from causing interference [3,154,155]. Shielding is achieved by blocking or attenuating EM waves through different mechanisms (as shown in Figure 6), such as absorption, reflection, and multiple reflection. Therefore, enhancing the performance and efficiency of EMI-shielding materials has become increasingly essential for maintaining technological infrastructure and achieving public health goals.

##### EMI-Shielding Mechanisms

The ability of EMI-shielding materials to attenuate and reflect EM waves is referred to as EMI-shielding effectiveness (SE), which is expressed as follows [3]:

(4) $${SE}_{T}\left(dB\right)=10\;\log_{10}\left(\frac{P_{I}}{P_{T}}\right)=20\;\log_{10}\left(\frac{E_{I}}{E_{T}}\right)=20\;\log_{10}\left(\frac{H_{I}}{H_{T}}\right)$$

where P, E, and H represent the power intensity, electric field intensity, and magnetic field intensity, respectively. The I and T subscripts denote the incident and transmitted waves. Since three different mechanisms (reflection, absorption, and multiple reflection) contribute to the total shielding, the total shielding effectiveness (${SE}_{T}$) is given by:

(5) $${SE}_{T}\left(dB\right)={SE}_{R}+{SE}_{A}+{SE}_{M}$$

Reflection loss occurs due to the impedance mismatch between the space ($\eta_{0}$, e.g., air) and the shielding material (η). Impedance mismatch is defined as the ratio of the electric field to magnetic field, E/H [3]. This phenomenon, related to the interactions of charged particles and EM waves, can be calculated using:

(6) $${SE}_{R}=-10\;\log_{10}\left(\frac{\sigma_{T}}{16f\varepsilon_{0}\mu_{r}}\right)$$

where $\sigma_{T}$ ($\Omega^{-1}m^{-1}$) is total conductivity, $\mu_{r}$ is the relative permeability of space, and f represents the frequency (Hz). $\varepsilon_{0}$ denotes the permittivity of a vacuum ($\approx 8.85\times 10^{-12}F/m)$. According to Equation (6), for stable values of $\sigma_{T}$ and $\mu_{r}$, the reflection loss is inversely proportional to the frequency of radiation.

Absorption loss arises from the interactions between electric and magnetic dipoles of the EMI shield and EM waves, resulting in thermal loss [2]. The amplitude of EM waves decreases exponentially as they pass through the shield [150]. This mechanism is a function of the skin depth parameter, defined as the depth at which the EM field intensity decreases to 1/e or 37% of the initial value, as expressed in Equation (7).

(7) $${SE}_{A}=20\;\log e^{t/\delta }=8.68\frac{t}{\delta }=8.68t\sqrt{{\pi f\sigma }_{T}\mu_{r}}$$

where t is the thickness; $\sigma_{T}$ ($\Omega^{-1}m^{-1}$) is the total conductivity; $\mu_{r}$ is the relative permeability of space; and f is the frequency (Hz). According to the equation, ${SE}_{A}$ increases with increasing thickness and decreasing skin depth. The skin depth (δ) is dependent upon the frequency (f), permeability (μ), and conductivity (σ) and can be calculated using:

(8) $$\delta ={\left({\pi f\sigma }_{T}\mu_{r}\right)}^{-1/2}$$

High electrical conductivity, dielectric permittivity, and magnetic permeability enhance absorption loss through ohmic loss, dielectric loss, and magnetic loss, respectively [151].

For thin shields (t<δ), multiple reflections occur when the EM waves reflect multiple times between boundaries. This can be calculated as follows:

(9) $${SE}_{M}=20\;\log_{10}\left(1-e^{\frac{-2t}{\delta }}\right)$$

The contribution of ${SE}_{M}$ can be neglected when δ≪t or ${SE}_{A}>15$.

#### 2.2.2. Advancements in Developing MXene Polymer Nanocomposites with High Dielectric Property

**Figure 7:**
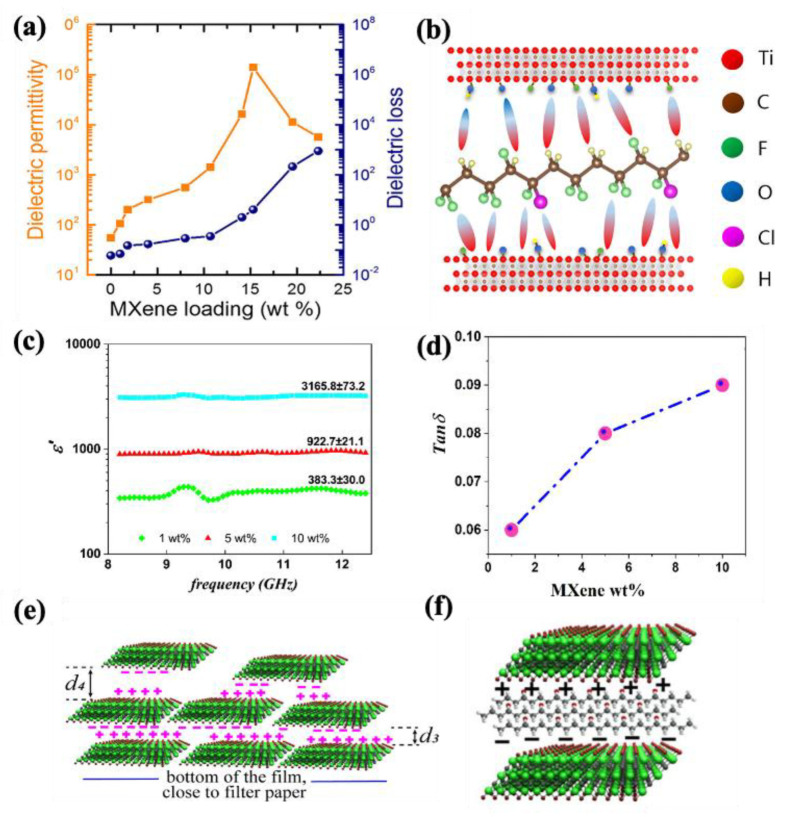
(**a**) Dielectric permittivity and dielectric loss of P[VDF-TrFE-CFE]–MXene nanocomposites as a function of MXene loadings at 1 kHz; (**b**) schematic illustration of the formation of dipoles between MXene surface and polymer backbone [157]. Copyright 2018, American Chemical Society. (**c**) Dielectric constant and (**d**) dielectric loss of PVA–MXene nanocomposites prepared by the VAF processing method, (**e**) microstructure of the sample prepared by the VAF method, and (**f**) schematic illustration of the nanocapacitor formation within the nanocomposite [122]. Copyright 2019, American Chemical Society.

Depending on the final applications, the requirement for dielectric properties can be different. For instance, an interconnected (but not fully percolated) MXene network is crucial to prevent conduction leakage in dielectric materials [156]. Conversely, for EMI shielding, concentrations higher than the percolation threshold can effectively reduce EM waves through absorption [144]. For energy storage, Tu et al. [157] incorporated Ti_3_C_2_T_x_ MXene into the poly (vinylidene fluoride–trifluoro-ethylene–chlorofluoroethylene) (P[VDF-TrFE-CFE]) polymer to improve dielectric performance. As shown in Figure 7a, a 10 wt% loading of MXene increased the dielectric constant by 25 times, while the dielectric loss increased from 0.06 to 0.35, only five times. This behavior was attributed to the large surface area of MXene, which facilitated the formation of microscopic dipoles and charge accumulation at the MXene–polymer interface (Figure 7b).

In another study performed on PVA/MXene nanocomposites, Mirkhani et al. [122] utilized two different methods, solution casting and vacuum-assisted filtration (VAF), to prepare nanocomposites with varying MXene loadings. They successfully achieved an outstanding dielectric performance with the addition of 10 wt% MXene using the VAF method, with a dielectric constant of 3166 and a tan δ of 0.09 (as shown in Figure 7c,d). This exceptional improvement was attributed to the high electrical conductivity of MXene, its uniform dispersion in the VAF method, and the resulting nacre-like structure. As depicted in Figure 7e, the penetration of PVA chains between MXene flakes created a nanocapacitor structure (Figure 7f), where the PVA layers acted as nanodielectrics between the MXene electrode layers. Table 4 provides a summary of studies on the dielectric properties of MXene–polymer nanocomposites, highlighting their potential for diverse electronic applications.

### 2.3. Mechanical Properties

The multilayered structure and strong M-C and M-N bonds in MXenes contribute to their exceptional mechanical properties, making them highly effective for electronics. Compared to more established materials like graphene, MXenes exhibit a superior tensile stiffness of 570 MPa and a Young’s modulus of 333 GPa [168]. Efforts to synthesize MXene nanosheets with minimal thickness and defects while maintaining their mechanical integrity have shown promising results. For instance, Rong et al. [169] employed an improved minimally intensive layer delamination (MILD) method to produce 0.98 nm-thick MXene nanosheets, achieving an impressive tensile strength of 15.4 GPa and a Young’s modulus of 483.5 GPa.

Surface functional groups in MXenes further enhance their compatibility with polymer chains, resulting in enhanced mechanical performance [170]. Zhang et al. [171] demonstrated this by incorporating 0.75 wt% MXene into ultrahigh molecular weight polyethylene (UHMWPE). Despite MXene’s hydrophilic nature, it increased the tensile strength of the composite to 39.65 MPa. Furthermore, the incorporation of MXene into a UHMWPE polymer matrix had a positive effect on reducing the friction coefficient from 0.186 for pure polymer to 0.128 for the nanocomposite sample with 2 wt% MXene.

The improvements are even more pronounced in polar polymers such as PVDF, PVA, PU, and EP [172,173,174,175]. For example, Zhi et al. [175] reported that adding 0.5 wt% MXene to a PU matrix resulted in a remarkable 70% increase in yield strength, increasing from 1.3 MPa in the pure PU to over 2.2 MPa in the nanocomposite.

In summary, compared to other 2D materials such as graphene and TMDCs, MXenes offer a unique combination of properties that make them particularly promising for advanced electronic applications [176,177]. Table 5 provides a comprehensive comparison between MXenes, graphene, and TMDCs, highlighting the distinct advantages of MXenes over their counterparts. The simultaneous presence of multiple desirable features within a single nanomaterial positions MXenes at the forefront of multifunctional material development.

## 3. Multifunctional MXene Materials with the Primary Application of Energy Storage

**Figure 8:**
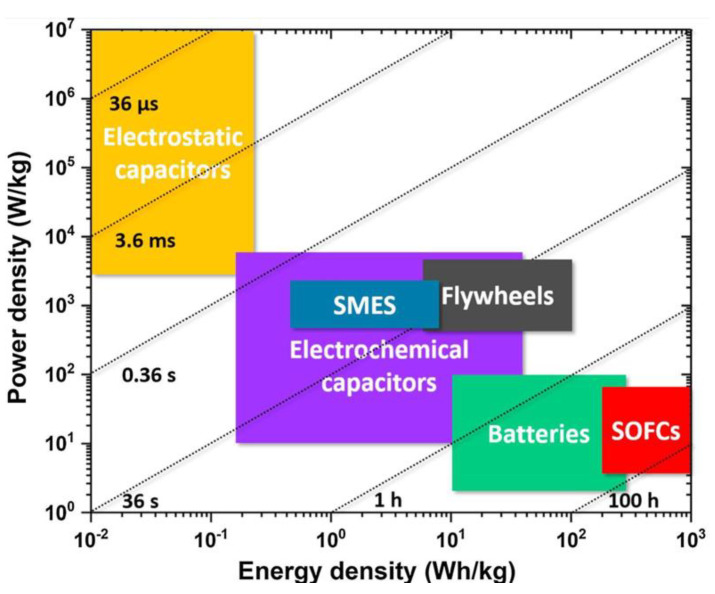
Ragone plot illustrating power density versus energy density, highlighting the performance of various energy storage systems [199]. Copyright 2018, Elsevier.

Modern industrial growth and the increasing demand for renewable energy sources have driven scientific progress in the development of high-efficiency energy storage systems [145,189]. Batteries, supercapacitors, and capacitors (electrostatic capacitors) are among the most commonly used energy storage devices, each offering unique properties tailored to specific applications [190]. Figure 8 illustrates the Ragone plot, comparing these devices based on their energy density and power density [191].

Each device type has distinct advantages and limitations. Batteries, characterized by a high energy density and a relatively low power density, are well-suited for applications requiring long-term energy supply, such as power electronics, mobility solutions, and grid storage [17,34]. In contrast, supercapacitors and electrostatic capacitors, which feature high power density but lower energy density, excel in applications where rapid energy discharge is critical, such as electric power transmission systems [36,192,193]. Enhancing the performance of polymer nanocomposites in these energy storage devices often involves incorporating nanofillers into polymer matrices to address performance limitations. Among the various nanofillers, different types of MXenes have gained significant attention due to their multifunctional properties. These include excellent electrical and electronic properties, a layered structure, large surface area, low energy barriers for electron transfer, and short ion-diffusion pathways [51,194,195,196].

The following sections review the role of MXenes in key components of energy storage devices, including their use as electrodes and electrolytes in batteries, electrolytes in supercapacitors, and dielectric materials in electrostatic capacitors [27,191,197,198]. While many studies have focused on the specific enhancements within individual devices, this review highlights research investigating the multifunctional performance of MXenes across diverse applications.

### 3.1. Batteries

According to previous sections, MXenes demonstrate exceptional potential for charge storage applications due to their unique layered structure, large surface area, excellent electrical conductivity (~24,000 S/cm for Ti_3_C_2_T_x_), hydrophilic nature, and rich surface chemistry [200]. These attributes make MXenes highly effective as advanced materials for electrodes and electrolytes in various battery systems, including metal-ion, metal–sulfur, and metal–oxygen batteries [201,202]. Current research emphasizes improving key performance metrics such as Coulombic efficiency, cyclic stability, dendrite suppression, and mechanical flexibility in advanced electrode materials. Similarly, the development of high-ionic-conductivity electrolytes is essential for enabling advanced technologies like wearable electronics. Beyond free-standing pure MXene films, nanocomposites that integrate MXenes with polymer matrices offer a synergistic combination of MXene’s superior properties with the mechanical flexibility and chemical stability of polymers. This combination strategy also enhances other functionalities and introduces new opportunities for expanding applications beyond energy storage systems [42,203,204,205]. The applications of polymer–MXene nanocomposites in batteries are primarily divided into two critical components: electrodes and electrolytes, which are detailed in the following sections.

#### 3.1.1. Electrodes

**Figure 9:**
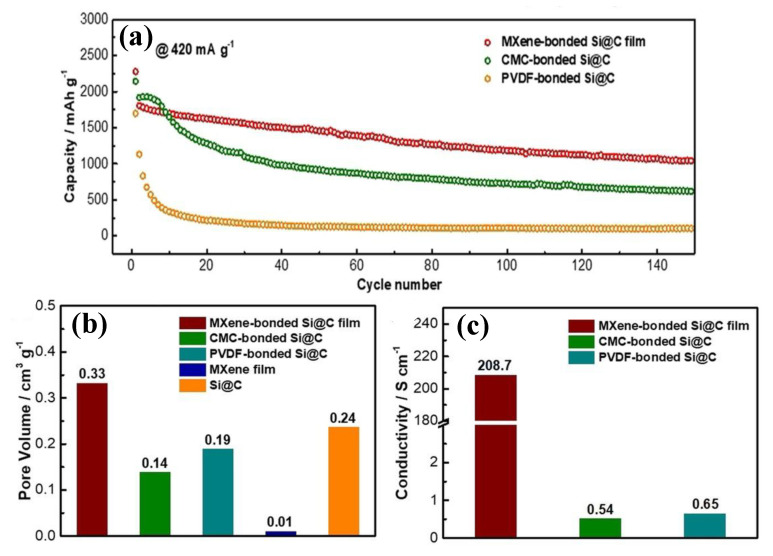
Comparison between lithium-ion performance composed of MXene-bonded Si@C film with CMC-bonded and PVDF-bonded Si@C electrodes: (**a**) rate capability (or stability), (**b**) pore volume, and (**c**) conductivity [206]. Copyright 2019, Wiley-VCH.

Zhang et al. [206] utilized MXene as a multifunctional binder in flexible silicon Si@C electrodes, promising anode materials for lithium-ion batteries (LIBs). As illustrated in Figure 9, their study highlighted several advantages of MXene compared to traditional binders such as carboxymethylcellulose sodium (CMC) or PVDF. These advantages included improved rate capability due to increased electrode conductivity, enhanced specific capacitance, superior flexibility, and excellent stability. The 3D porous MXene framework played a pivotal role by accommodating significant volume changes during cycling, thereby contributing to long-term performance.

**Figure 10:**
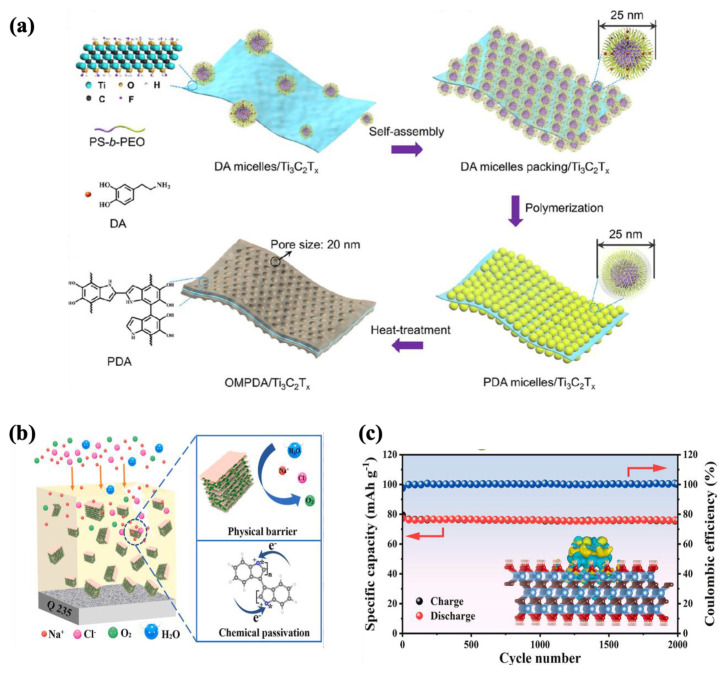
(**a**) Preparation of OMPDA/Ti_3_C_2_T_x_ composite using in situ polymerization of dopamine and subsequent heat treatment [208]. Copyright 2020, American Chemical Society. (**b**) Schematic indicating anti-corrosion mechanism in Pind–MXene composite (*, reactive positions) and (**c**) the performance of Pind–MXene composite for long-term cycling [209]. Copyright 2023, Elsevier.

Flexible multilayered electrodes have emerged as a promising way to harness the distinct advantages of individual layers, resulting in synergistic improvements in overall performance [207]. Li et al. [208] developed a sandwich-structured composite of ordered mesoporous polydopamine (OMPDA)–Ti_3_C_2_T_x_ as a high-performance anode material for rechargeable batteries (as shown in Figure 10a). This innovative structure was fabricated through in situ polymerization of dopamine on the MXene surface using DA-PS-b-PEO micelles as a soft template, followed by heat treatment. The resulting composite electrodes exhibited an impressive reversible capacitance of 1000 mAh/g at 50 mA/g and showed excellent stability, retaining their performance over 200 cycles.

Among polymer-based electrodes, conductive polymers like poly(3,4-ethylenedioxythiophene) (PEDOT), polyindole (Pind), and polyaniline (PANI) are highly advantageous due to their excellent electrical conductivity, making them ideal for energy storage applications [210,211,212,213]. A dual-functional Pind–MXene composite was developed through a straightforward polymerization method for use in both energy storage and corrosion protection (Figure 10b). The composite electrode delivered a notable specific capacitance of 118 mAh/g and an exceptional cycle stability, retaining 98.2% of its capacity after 2000 cycles with a coulombic efficiency of 100% (Figure 10c). Additionally, when applied as an anticorrosive coating on epoxy, the composite achieved a remarkably low corrosion rate of 9.17 × 10^−6^ mm/a and an impressive corrosion inhibition efficiency of 99.72% [209]. It is worth mentioning that the research on the usage of MXene in batteries has mostly focused on the electrolyte component.

#### 3.1.2. Electrolytes

**Figure 11:**
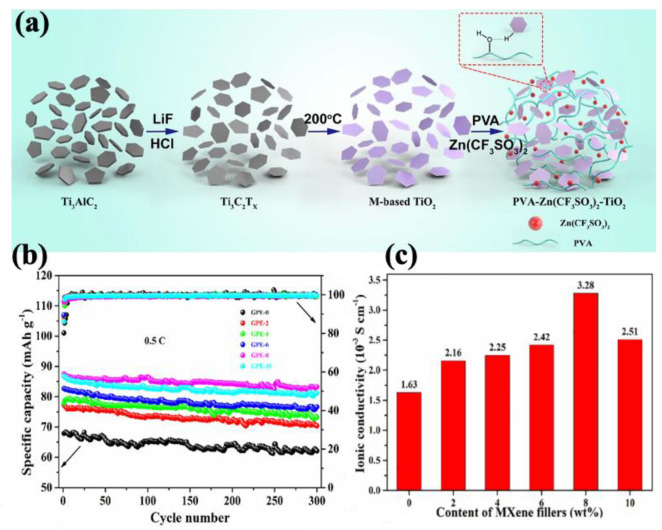
(**a**) Schematic illustrating the fabrication process of the PVA–MXene–TiO_2_ gel electrolyte [215]. Copyright 2022, Elsevier. (**b**) Long-term cycle performance and (**c**) ionic conductivity of (Na_3_V_2_(PO_4_)_3_/GPE/Na) battery [216]. Copyright 2021, Elsevier.

Chen et al. [214] developed a solid polymer electrolyte composed of P(VDF-HFP) filled with PMMA-grafted MXenes for zinc-ion batteries (ZIBs). The uniform dispersion of MXenes resulted in a high ionic conductivity of 2.69 × 10^−4^ S/cm at room temperature. Beyond its primary function, this innovative design mitigated side reactions, such as the hydrogen evolution reaction (HER), suppressed anode dendrite formation, and maintained performance across a wide temperature range, from −35 °C to 100 °C. Considering the importance of flexibility in wearable energy storage devices, Liu et al. [215] fabricated a gel polymer electrolyte (GPE) based on PVA–MXene–TiO_2_ through a one-step hydrothermal reaction (illustrated in Figure 11a). The hybrid gel electrolyte exhibited a high capacity of 216 mAh/g after 115 cycles, as well as enhanced mechanical properties and an effective self-healing ability. In another study conducted by Wang et al. [216], a GPE was fabricated using a combination of PVDF-HFP, PMMA, and Ti_3_C_2_T_x_ MXene through the solution-casting method for sodium-ion batteries (SIBs). According to Figure 11b,c, the GPE containing 8 wt% Ti_3_C_2_T_x_ MXene achieved a high ionic conductivity of 3.28 × 10^−3^ S/cm and retained 95.1% of its capacity after 300 cycles at 0.5 C in a (Na_3_V_2_(PO_4_)_3_/GPE/Na) battery. Beyond its primary functions, the incorporation of MXene also effectively suppressed sodium dendrite growth and markedly enhanced thermal stability.

**Figure 12:**
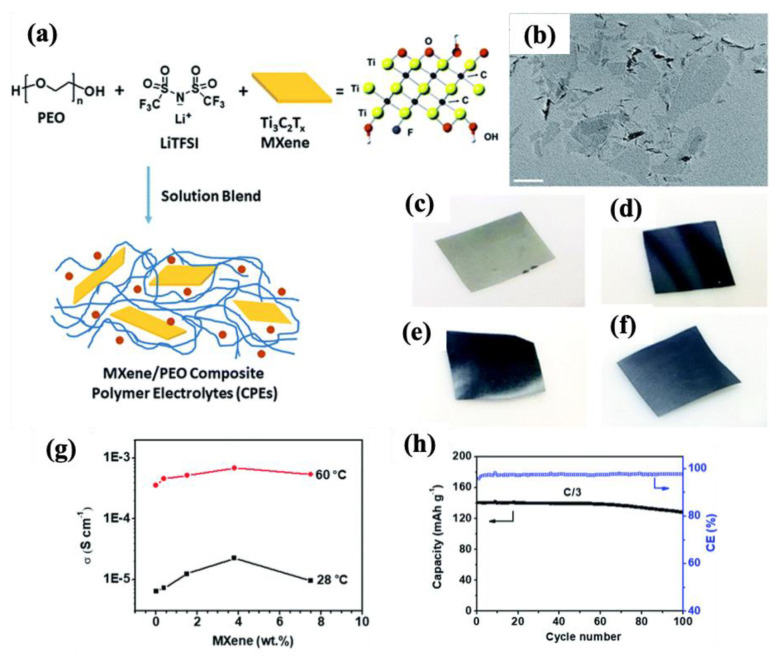
(**a**) Preparation process of MXene-based nanocomposites, (**b**) TEM image of the synthesized few-layer MXene with scale bar of 100 nm, (**c**–**f**) photographs of flexible, lightweight polymer nanocomposite membranes, (**g**) ionic conductivity of nanocomposites as a function of MXene content at 28 °C and 60 °C, and (**h**) discharge capacity and coulombic efficiency of LiFePO_4_|PEO_20_-LiTFSI-MXene^0.02^|Li system as a function of cycle number [35]. Copyright 2018, Royal Society of Chemistry.

Pan et al. [35] prepared a flexible, lightweight, and multifunctional all-solid-state LIB based on a PEO/LiTFSI complex containing different contents of Ti_3_C_2_T_x_ MXene using a green aqueous solution-blending process (Figure 12a–f). The high surface area and hydrophilic nature of MXene, which led to the uniform dispersion of MXene within the polymer nanocomposites, resulted in a significant enhancement in different functionalities, such as ionic conductivity, rate capability, and stability of the developed LIB. For example, according to Figure 12g, the ionic conductivity of the nanocomposite achieved its highest value of 2.2 × 10^−5^ S/m at 28 °C with the addition of 3.6 wt% MXene. Furthermore, it can be seen in Figure 12h that, with the incorporation of 1.5 wt% MXenes (PEO_20_-LiTFSI-MXene^0.02^ sample), the LIB showed a much higher retention capacity of 91.4% and an excellent coulombic efficiency of >97% after 100 cycles.

**Figure 13:**
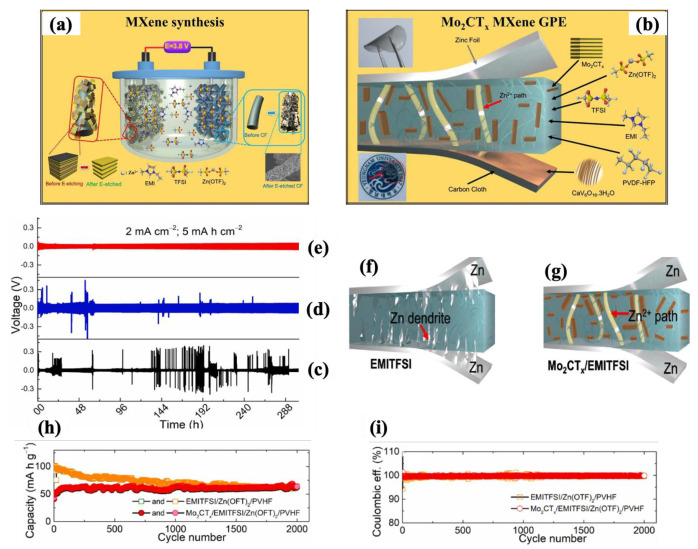
(**a**) Electrochemical method of MXene synthesis, (**b**) schematic illustration of full-cell of CaV_6_O_16_⋅3H_2_O//Mo_2_CT_x_/EMITFSI/Zn(OTF)_2_/PVHF//Zn, galvanostatic plating/stripping curves of GPEs of (**c**) EMITFSI//Zn(OTF)_2_/PVHF, (**d**) Mo_2_CT_x_/Zn(OTF)_2_/PVHF, and (**e**) Mo_2_CT_x_/EMITFSI//Zn(OTF)_2_/PVHF at 2 mA cm^−2^ current density, schematic illustrating the possible dendrite formation in the (**f**) Mo_2_CT_x_/Zn(OTF)_2_/PVHF and (**g**) EMITFSI//Zn(OTF)_2_ GPEs, (**h**) cyclic stability and (**i**) coulombic efficiency of CaVO//EMITFSI/Zn(OTF)_2_//Zn, and CaVO//Mo_2_CT_x_/EMITFSI/Zn(OTF)_2_//Zn cells at a current density of 0.14 A/g [217]. Copyright 2023, Elsevier.

Kumar et al. [217] performed a thorough investigation on the use of Mo_2_CT_x_ MXene in ZIBs. As seen in Figure 13a, they first synthesized MXene through the electrochemical etching method using a 1-ethyl-3-methylimidazolium bis(trifluoromethyl sulfonyl)imide (EMITFSI) and zinc trifluoromethane sulfonate (Zn(OTF)_2_) battery electrolyte. Then, they prepared a gel polymer electrolyte (as shown in Figure 13b) composed of Mo_2_CT_x_/EMITFSI/Zn(OTF)_2_/poly(vinylidene fluoride-co-hexafluoropropylene) (PVHF) using solution mixing. Finally, they assembled a ZIB cell with CaV_6_O_16_⋅3H_2_O as the cathode and Zn as the anode. In comparison to EMITFSI/Zn(OTF)_2_/PVHF and Mo_2_CT_x_/Zn(OTF)_2_/PVHF, the prepared cell from the Mo_2_CT_x_/EMITFSI/Zn(OTF)_2_/PVHF electrolyte illustrated a high ionic conductivity and inhibited Zn dendrite formation over 300 h of continuous plating/stripping (as shown in the galvanostatic plating/stripping curves in Figure 13c–e). The schematic in Figure 13f,g displayed the possible dendrite formation on the zinc foils in the Mo_2_CT_x_/Zn(OTF)_2_/PVHF and EMITFSI//Zn(OTF)_2_ GPEs. According to Figure 13h,i, this limited dendrite formation resulted in an impressive durability of 99% and an outstanding coulombic efficiency of approximately 100% over 2000 cycles in the assembled ZIB cell made with Mo_2_CT_x_/EMITFSI/Zn(OTF)_2_/PVHF.

### 3.2. Supercapacitors

Supercapacitors (SCs) are another common energy storage device valued for their high-power density and rapid charge–discharge rates, making them superior to batteries in applications requiring quick energy delivery [12,37,218]. However, their relatively low energy density restricts their use in certain applications and, thus, drives substantial research efforts to overcome this drawback [219]. Based on their energy storage mechanisms, SCs are classified into three main types: (1) pseudocapacitors (PCs), which store energy through rapid, reversible redox reactions occurring at the electrode–electrolyte interface; (2) electrochemical double-layer capacitors (EDLCs), which rely on the physical electrostatic adsorption of ions onto the surface of the electrodes, creating an electrostatic double layer for energy storage; and (3) hybrid capacitors (HCs), which combine both pseudocapacitive and double-layer mechanisms to achieve improved energy storage performance [220,221,222,223,224,225]. MXenes are appropriate for supercapacitors, particularly the electrode component, due to their high electrical conductivity, large surface area, and tunable surface chemistry [221,222,223]. These attributes enable efficient ion transport, improved charge storage, and enhanced electrochemical performance [32,226]. Therefore, numerous studies have focused on exploiting MXenes in supercapacitors to bridge the gap between high power density and energy density, advancing the development of next-generation energy storage technologies in various applications, such as electronics [38,227,228].

**Figure 14:**
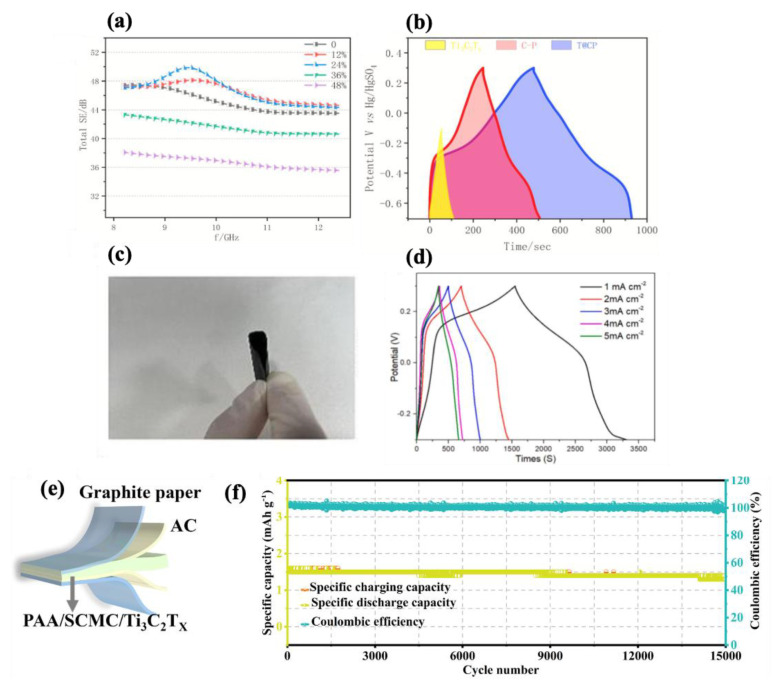
(**a**) SE_T_ plots of T@CP at different *C*–P contents, (**b**) GCD curves for pure MXene (Ti_3_C_2_T_x_), CNT/PANI (C-P), and T@CP [232]. Copyright 2021, Elsevier. (**c**) Picture of MXene–CNF–PANI composite, (**d**) GCD of MXene–CNF–PANI composite film at different scan rates [233]. Copyright 2022, Elsevier. (**e**) Schematic illustration of the supercapacitor, and (**f**) cyclability and coulombic efficiency at 1 A g^−1^ [234]. Copyright 2024, Elsevier.

Hybridizing MXene with other nanomaterials or integrating it with other polymers is a promising strategy to achieve multifunctional properties. This strategy leverages the unique attributes of each component, enhancing electrochemical performance while introducing additional functional capabilities [1,229,230,231]. For instance, Fang et al. [232] developed a bifunctional MXene (Ti_3_C_2_T_x_)-based composite for both electrode and EMI-shielding applications by incorporating a multi-walled carbon nanotube (MWCNT) and PANI into Ti_3_C_2_T_x_. As shown in Figure 14a,b, the Ti_3_C_2_T_x_-CNT/PANI composite (T@CP) demonstrated a substantial total EMI-shielding effectiveness (SE_T_) of 49.8 dB and specific capacitance of 2134.5 mF cm^−2^ at a scan rate of 2 mV/s. This performance markedly outperformed pure Ti_3_C_2_T_x_, which achieved an SE_T_ of 45.3 dB and a specific capacitance of 414.3 mF cm^−2^ under the same conditions. Using a simple vacuum filtration technique, Yuan et al. [233] fabricated an eco-friendly flexible electrode material composed of an MXene–cellulose nanofiber (CNF)–PANI composite (Figure 14c). The resulting hybrid films exhibited enhanced electrochemical and mechanical properties. For instance, the material exhibited a high areal specific capacitance of 2935 mF cm^−2^ at a current density of 1 mA cm^−2^ and retained 94% of its capacitance after 2000 cycles at 10 mA cm^−2^ (Figure 14d). The improved performance was attributed to the synergistic effects of MXene and PANI, which boosted electrochemical activity, while CNF increased the interlayer spacing of MXene, facilitating better ion diffusion in the electrolyte. In another study on cellulose-based materials, researchers designed multifunctional hydrogels by incorporating sodium carboxymethyl cellulose (SCMC) and MXene (Ti_3_C_2_T_x_) into a polyacrylic acid (PAA) (Figure 14e). These hydrogels demonstrated promising performance as flexible, wearable strain sensors. For example, as shown in Figure 14f, the hydrogels exhibited excellent cyclability, enduring over 15,000 charge–discharge cycles, highlighting their potential for supercapacitor applications. Additionally, they achieved gauge factors (GF) = 5.79 in the strain range of 0–700%, GF = 14.0 in the range of 700–900%, and GF = 40.36 for 900–1000% [234].

**Figure 15:**
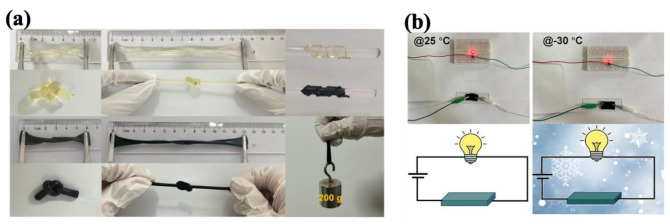
(**a**) Optical images indicating the high stretchability of the GP-Fe electrolyte (the pale-yellow strip) and MPGP-Fe electrodes (the black strip), and (**b**) the performance of the device at two different temperatures of 25 °C and −30 °C [235]. Copyright 2024, Elsevier.

Han et al. [235] developed a flexible hydrogel-based electrode by integrating MXene–PANI (MP), Fe^3+^, and phytic acid into a gelatin–polyacrylamide (GP) hydrogel scaffold for supercapacitors used in various electronic devices. The fabricated all-gel supercapacitors utilized MPGP-Fe_y_ (y, representing the molar concentration of Fe^3+^) as the electrodes and GP-Fe_y_ as the electrolyte. The system achieved a maximum specific capacitance of 847 mF cm^−2^, a significant energy density of 71.8 μWh cm^−2^, and a capacitance retention of 95% after 5000 cycles. The addition of phytic acid played a crucial role by lowering the freezing point of the supercapacitor components, encompassing both the electrode materials and the electrolyte layer. As illustrated in Figure 15a,b, this hydrogel-based system demonstrated a wide operating temperature range (−30 to 90 °C), along with excellent flexibility and stretchability as other functionalities, making it highly suitable for use in various electronic devices.

**Figure 16:**
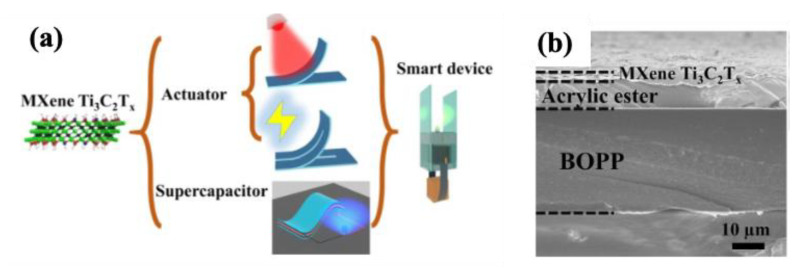
(**a**) Schematic illustration of the multifunctionality of bilayer MXene–BOPP composite film as actuators and supercapacitors for smart devices and (**b**) cross-sectional SEM image of the MXene/BOPP film [237]. Copyright 2022, Elsevier.

Beyond the fabrication of nanocomposites with multiple polymers or nanomaterials, designing multilayered composites offers a macroscopic strategy to harness the unique properties of each layer. This strategy not only enhances overall performance but also introduces new functionalities [236]. Wang et al. [237] developed a bilayer composite film using a “one-for-all” technique, combining monolithic MXene and biaxially oriented polypropylene (BOPP) for use in actuators and supercapacitors. The electrodes fabricated from this composite exhibited an impressive aerial capacitance of 358.2 mF cm^−2^ at 0.1 mA cm^−2^. Furthermore, the system functioned as an actuator capable of responding to both light and electrical stimuli, achieving a maximum bending curvature of 1.51 cm^−1^ with no obvious decay after 600 cycles. The inclusion of the BOPP layer significantly enhanced flexibility and mitigated the brittleness of the MXene layer. As shown in Figure 16a,b, the dual functionality makes this multilayered system a promising candidate for advanced applications, such as smart devices and soft robotics.

Table 6 summarizes recent studies on conductive polymer-based nanocomposites, highlighting their primary applications in electrode materials for supercapacitors, specifically pseudocapacitors.

### 3.3. Electrostatic Capacitors

**Figure 17:**
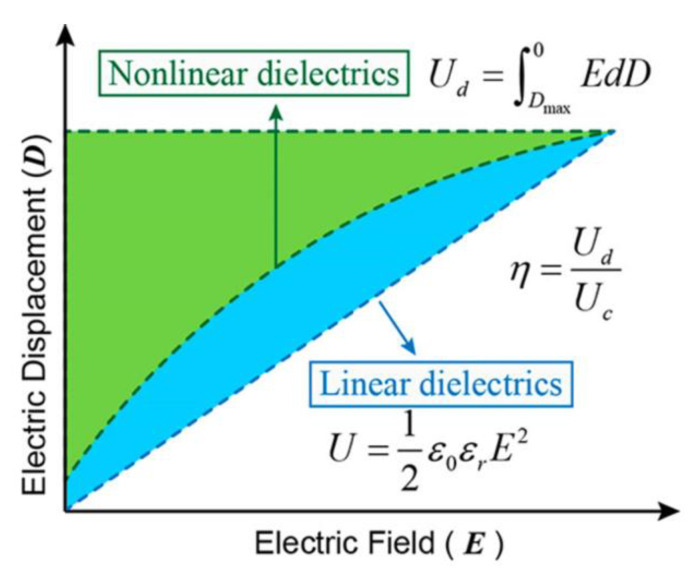
D-E hysteresis loop for calculating the energy density and energy loss density [191].

Electrostatic capacitors are among the essential energy storage devices due to their high power density and rapid charge–discharge capabilities (as shown in Figure 8), making them suitable for applications such as electric vehicles, power transmission, and renewable energy systems like wind power [244]. However, they suffer from low energy density. As a result, a key challenge in designing electrostatic capacitors is achieving both high energy density and high power density. The energy density of electrostatic capacitors is controlled by the dielectric material placed between two electrodes with opposite static charges. The displacement of charge carriers under an electric field within the dielectric material, which is known as the polarization process, can store energy [36]. The energy density ($U_{e}$) can be determined by integrating the electric field (E) across the electric displacement (D) axis in the discharge loop (Figure 17, green area), as described by Equation (10) [146]:

(10) $$U_{e}=\int_{D_{r}}^{D_{max}}EdD$$

where $D_{r}$ and $D_{max}$ are the remnant electrical displacement and maximum dielectric displacement, respectively.

In linear dielectrics, such as most polymeric-based composites, there is no energy loss, and the electric displacement is proportional to the applied electric field. This relationship is described by $D=\varepsilon_{0}\varepsilon_{r}E_{b}$, where the dielectric permittivity, $\varepsilon_{r}$, remains constant [245]. Therefore, the energy density can be calculated as follows:

(11) $$U_{e}=\frac{1}{2}\varepsilon_{0}\varepsilon_{r}E_{b}^{2}$$

where $\varepsilon_{0}$ and $E_{b}$ are the dielectric permittivity in a vacuum and the breakdown strength, respectively. It is worth mentioning that, during the discharging process, a portion of the stored energy is dissipated as heat ($U_{loss}$, illustrated as the blue area in Figure 17) [245]. Therefore, the energy storage efficiency, η, can be expressed as:

(12) $$\eta =\frac{U_{discharge}}{U_{charge}}=\frac{U_{e}}{U_{e}+U_{loss}}$$

The key parameters that are essential for dielectric materials include: (1) the dielectric constant, which measures a material’s ability to store energy, with higher values corresponding to increased energy storage capacity; (2) dielectric loss, representing the energy dissipated as heat, which must be minimized to enhance capacitor efficiency and longevity; (3) breakdown strength is the maximum electric field a material can withstand without becoming conductive, which crucial for dielectric materials, as energy density is a quadratic function of the breakdown strength (refer to Equation (11)). However, it should be noted that higher breakdown strength often correlates with a lower dielectric constant, necessitating a careful balance to optimize energy density; (4) thermal conductivity, essential for efficient heat transfer to prevent accelerated aging during charge–discharge cycles; (5) mechanical properties, important for durability, longevity, and resistance to deformation under electrical stress and thin-film manufacturing, which is critical for cost-effective, large-scale production; and (6) thermal stability, important for exposing dielectric materials to elevated temperatures due to electrical currents and external environmental conditions [191,246,247,248].

Dielectric materials used in electrostatic capacitors are typically ceramic or polymeric based. Ceramic-based dielectrics possess advantages like a high dielectric constant, stiffness, and excellent thermal stability. However, their low breakdown strength, poor flexibility, and difficult processability limit their practical applications. In contrast, polymer-based dielectrics are a reliable choice for this type of application, owing to their high breakdown strength and superior processability.

BOPP films, a linear dielectric, are the most commercially available polymer dielectrics for industrial applications, primarily due to their high breakdown strength (500–600 MV/mm) [146]. However, its low dielectric constant (~2.2) restricts its broader applicability. Over recent decades, other polymers such as PVDF, polyurethane (PU), polyimides (PI), acrylate resin, and PVA indicate their potential to be promising alternatives for polymer-based dielectrics [192,244]. Among them, PVDF, a normal ferroelectric polymer, stands out due to its relatively high dielectric constant, making it a strong candidate for advanced dielectric capacitors. Extensive research has focused on strategies ranging from the nanoscale, such as incorporating various nanofillers, to achieve a balance of high dielectric constant, high breakdown strength, and low dielectric loss. Barium titanate (BaTiO_3_) is a widely used traditional ceramic filler for enhancing dielectric properties. However, achieving a high dielectric constant typically requires a high loading of BaTiO_3_ (~30 wt%), which can negatively impact other properties, such as dielectric loss, flexibility, and the processability of polymer dielectrics. While BaTiO_3_ is effective in increasing the dielectric constant, its drawbacks limit its versatility. In contrast, incorporating MXenes as highly conductive nanofillers offers a unique balance between dielectric performance and mechanical properties, particularly at a low filler concentration [249,250]. This, along with their ability to promote multifunctionality, makes MXenes promising candidates for capacitor applications. This section provides a summary of recent advancements in MXene–polymer nanocomposites, focusing on their multifunctional applications, with electrostatic capacitors as the primary focus [147,189,191].

**Figure 18:**
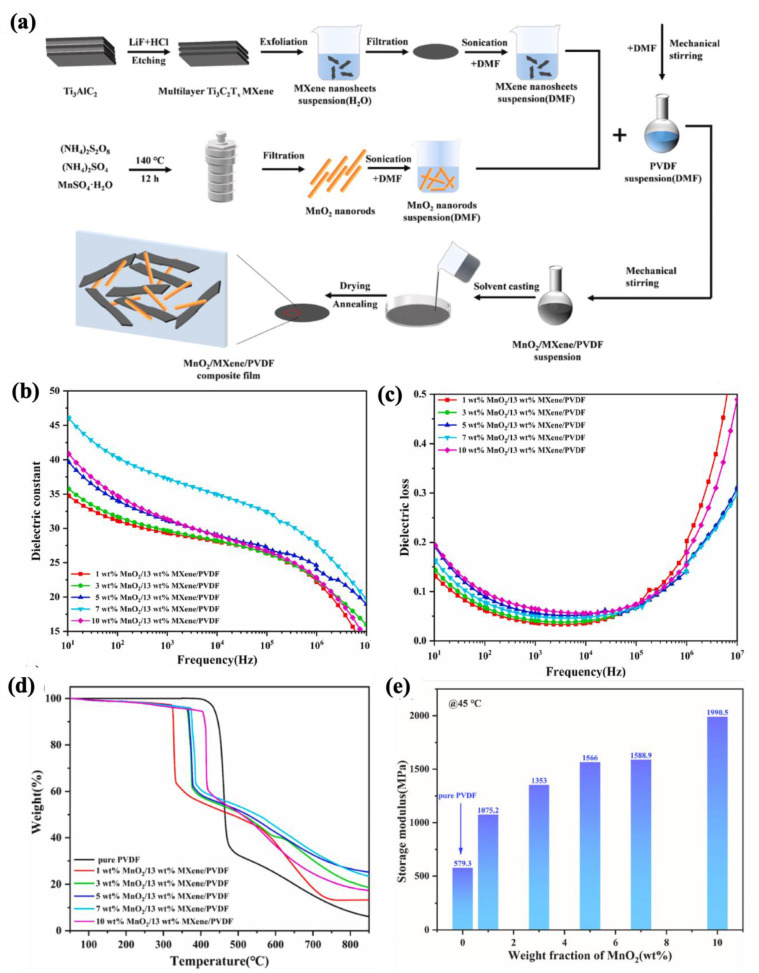
(**a**) Schematic illustration of PVDF– MXene–MnO_2_ nanocomposite films, (**b**) dielectric constant and (**c**) dielectric loss of nanocomposite films as a function of frequency, (**d**) TGA curves, and (**e**) storage modulus of pure PVDF and PVDF–MXene–MnO_2_ nanocomposite films [251]. Copyright 2024, Elsevier.

Cao et al. [251] prepared PVDF-based nanocomposites with hybrid nanomaterials of MXene and MnO_2_ using the solution casting method (Figure 18a). According to the dielectric properties (as shown in Figure 18b,c), they observed that the simultaneous usage of MXene and MnO_2_ led to an increase in the dielectric constant while decreasing the dielectric loss. For example, a nanocomposite sample containing 13 wt% MXene and 7 wt% MnO_2_ indicated a dielectric constant of 37.3 and a dielectric loss of 0.05 at a frequency of 1 kHz. They attributed these properties to improved interfacial polarization and the formation of micro-capacitors within the nanocomposites. Additionally, nanocomposites exhibited improved thermal and mechanical properties (Figure 18d,e) (e.g., a storage modulus of 1990.5 MPa for the nanocomposite film with 13 wt% MXene and 10 wt% MnO_2_, 3.44 times higher than PVDF) compared to pure PVDF.

**Figure 19:**
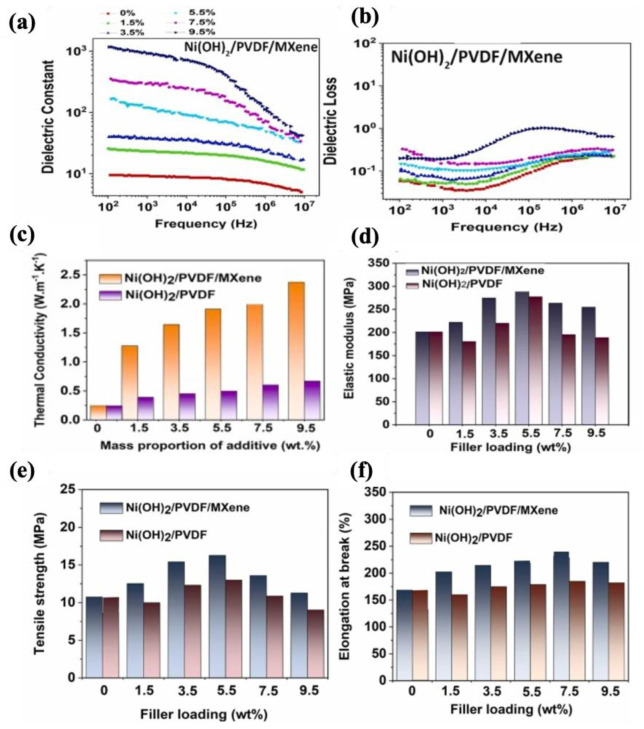
(**a**) Dielectric constant, (**b**) loss tangent of MXPN composite films, (**c**) out-of-plane thermal conductivity, (**d**) elastic modulus, (**e**) tensile strength, and (**f**) elongation at break of pure PVDF, nanocomposite films without MXene (NiPV) and with MXene (MXPN) [49]. Copyright 2024, Elsevier.

In another study on PVDF-based nanocomposites, Dizayee et al. [252] developed composites based on Ni(OH)_2_ and MXene (MXPN composites) using a solution mixing method to achieve a synergistic effect on improving dielectric, mechanical, and thermal properties. They used PEI to improve the interaction between Ni(OH)_2_ and MXene. Based on the dielectric performance in Figure 19a,b, the nanocomposite sample containing 9.5 wt% nanofiller exhibited a high dielectric constant of 1000 and a low loss tangent of 0.4 at 1 kHz, which were related to the formation of the nanohybrid structure within the nanocomposites. Moreover, significant increases in out-of-plane thermal conductivity (Figure 19c) and tensile properties (Figure 19d–f) were observed for MXPN composites with hybrid fillers compared to the composite films without MXene nanosheets.

**Figure 20:**
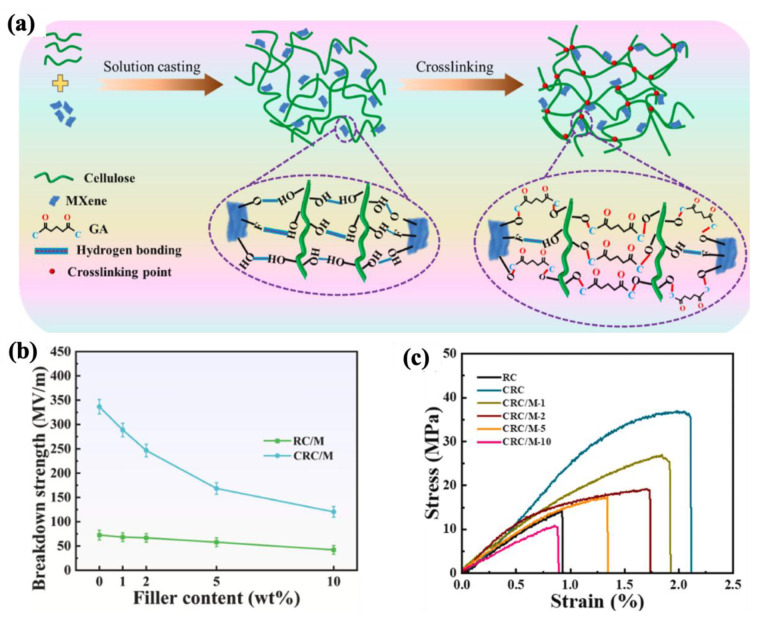
(**a**) Schematic illustration of the crosslinking reactions between polar groups of cellulose and MXene in the CRC/M composite films, (**b**) breakdown strength of CRC/M composite films as a function of MXene content, and (**c**) stress-strain curves of CRC/M composite films [253]. Copyright 2024, Elsevier.

Zhang et al. [253] developed crosslinked regenerated cellulose–MXene (CRC/M) composite films via glutaraldehyde (GA)-assisted crosslinking reactions with different loadings of MXene. According to their observations (Figure 20a), crosslinking reactions between the polar groups of cellulose polymer matrix and MXene functional groups led to a more integrated structure, decreasing defects. Consequently, it resulted in decreasing the dielectric loss and increasing the breakdown strength, as indicated in Figure 20b. For instance, the breakdown strength of the composite with 1 wt% MXene loading was 288.61 MV/m, with the energy density of 2.41 J/cm^3^, which was approximately 6.34-fold and 1.13-fold that of the neat RC film (0.38 J/cm^3^) and pure CRC film (2.12 J/cm^3^), respectively. However, it should be noted that these reactions had a negative effect on the dielectric constant. Moreover, the reduced hydrophilicity of these composites resulted in an improvement in the mechanical properties (Figure 20c).

Table 7 summarizes recent studies on MXene–polymer nanocomposites, highlighting their primary applications in electrostatic capacitors.

## 4. Multifunctional MXene Materials with the Primary Application of Sensing and Joule Heating

### 4.1. Theoretical Background on Sensing Mechanism

**Figure 21:**
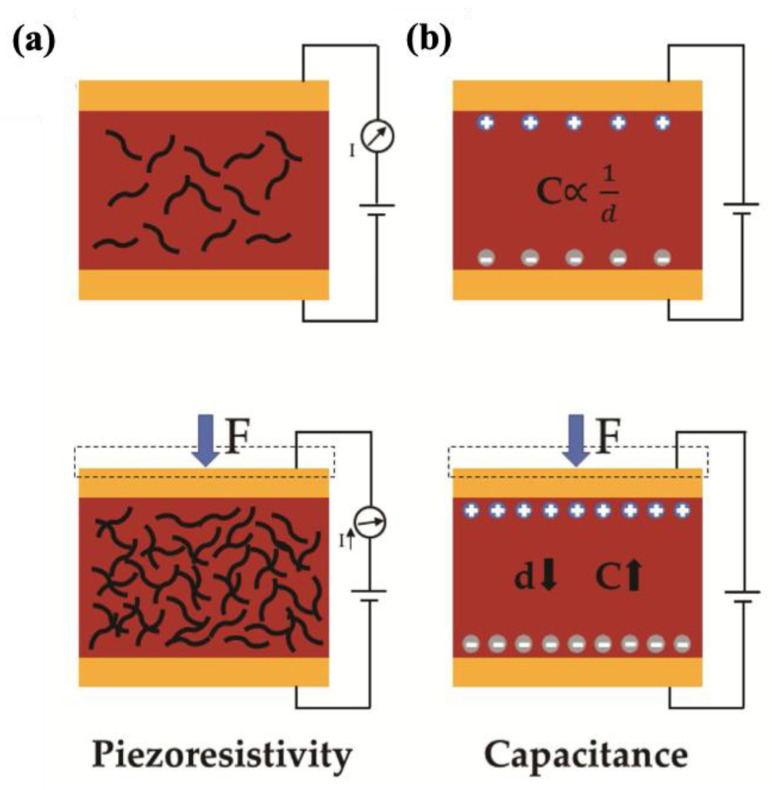
Mechanisms for deformation sensing: (**a**) piezoresistive and (**b**) capacitive-sensing phenomena [276]. Copyright 2020, Elsevier.

During the last decade, there has been an ascending trend in demand for multi-functional materials. More specifically, sensor devices have been requiring at least one more functionality concerning soft and wearable electronics, soft and intelligent robotics, artificial and electronic skins, and health monitoring devices [269,270,271,272,273]. Therefore, integrating sensory functionality within an engineered material can satisfy the essential need for the above-mentioned advanced industries. Basically, sensors are capable of detecting changes in external conditions, such as deformation, temperature, humidity, and chemical species, and reflect these changes into mainly electrical signals. For wearable detective devices, the main purpose is to detect and distinguish between human body motions and vocal activities. There are two types of electronic phenomena concerning the precise different motions and deformation modes, named piezoresistivity and capacitive sensing [273,274,275]. Figure 21 represents piezoresistivity and capacitive-sensing phenomena. In piezoresistive sensing, a composite, which is made of conductive fillers like carbon-based, metal-based, and transition metal carbides and nitrides (MAX phase and MXene), possesses a distinct electrical conductivity if the filler’s concentration falls at least around the percolation threshold. Applying compressive deformation moves the fillers closer to one another and makes them more interconnected, thereby decreasing the electrical resistance and enabling current flow through a closed electric circuit under constant voltage. On the other hand, stretching the same composite material leads to the destruction of the conductive fillers’ network, and the deterioration of electrical resistance increases accordingly. Capacitive-sensing phenomenon follows the general equation of capacity (Equation (13)), in which C is capacity, ε is dielectric constant, S is the electrode area, and d is the distance between two parallel electrodes. The opposite relationship between capacity and the distance between electrodes suggests that applying pressure results in a leap in the capacity. Based on the explanation so far, every deformation in both piezoresistive and capacitive sensors leads to a distinct change in electrical resistance or capacity, respectively, while the deformation mode and its extent can be quantified accordingly [276,277].

(13) $$C=e\frac{S}{d}$$

### 4.2. Different Sensors and Heaters Categories Based on Substrate’s Macrostructures

Different main applications of multifunctional MXene materials call for a distinct macrostructure to cope with the special needs of interest. For example, fabric substrates or spun fibers comply with the essential needs for knittability and/or wearability, as well as breathability, for wearable electronics applications. Hydrogel-based MXene materials are suitable for soft robotics and adaptability for electronic skin (E-skin) applications thanks to their excellent stretchability and possible self-healing. Other kinds of macrostructures, such as foams, freeze-dried mediums, and layered films, are useful for fabricating ultra-lightweight structures with enhanced EMI shielding and superior sensitivity. In the following subsections, MXene–polymer nanocomposites are categorized based on the above-mentioned macrostructure’s varieties for multifunctional sensory and/or Joule heating applications.

#### 4.2.1. Fabric-Based Multifunctional Sensors and Heaters

**Figure 22:**
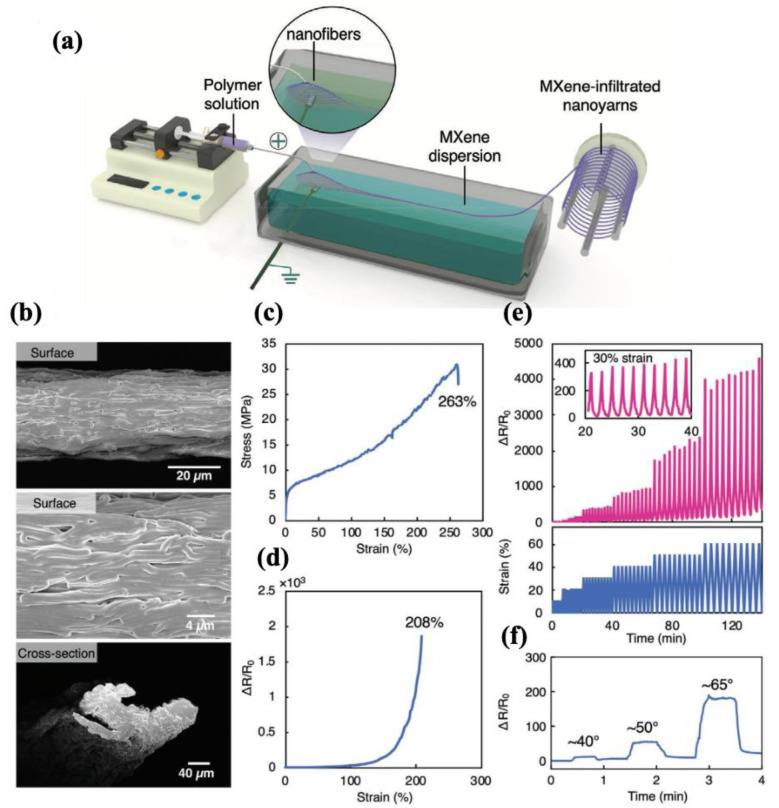
(**a**) Bath electrospinning procedure mechanism, (**b**) SEM images of MXene–PU nanoyarns with 19 wt% MXene, (**c**) stress-strain curve of MXene–PU nanoyarns, (**d**–**f**) piezoresistive responses of MXene–PU nanoyarns with 19 wt% MXene versus (**d**) strain, (**e**) time, and (**f**) bending angle [278]. Copyright 2020, Wiley-VCH.

**Figure 23:**
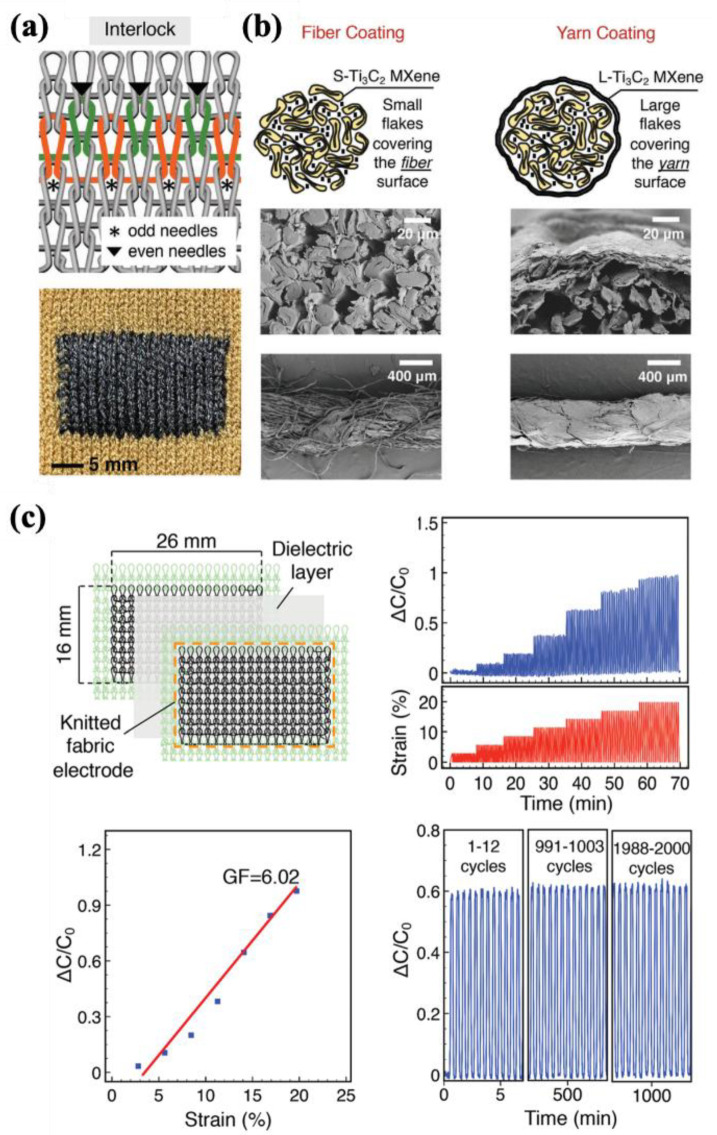
(**a**) Proposed knitting technique for MXene-coated cellulose fiber, (**b**) visualization of the optimized coating strategy, and (**c**) capacitive-sensing functionality of the knitted coated fabric electrode [279]. Copyright 2019, Wiley-VCH.

For wearable electronics and human body motion monitoring, flexibility, wearability, and breathability are essential factors where the choices of multifunctional sensory materials and fabrication processes are limited. Consequently, multifunctional electroactive knittable yarns and fabrics are of great interest for this purpose [273,278]. Levitt et al. [278] proposed one-step bath electrospinning, which can make Nylon and PU nanoyarns possessing an extraordinary Ti_3_C_2_T_x_ MXene uptake up to 90 wt%, and flexibility for stretchable electronics and body movement monitoring. This fabrication method offers resolution for the shortage in stretchability in the samples fabricated by fiber wet-spinning in an MXene bath or a polymer–MXene composite with up to 70 wt% MXene loading. Figure 22a demonstrates the bath electrospinning procedure in which electrospinning and wet spinning are combined to operate based on coagulation in an MXene bath. This technique allows the solution to effectively infiltrate nanoyarns for achieving a substantially high loading content, even in hydrophilic polymers like PU. The achieved stretchabilities for MXene–Nylon and MXene–PU nanoyarns are recorded as 43% and 263%, with the electrical conductivities of 1195 S/cm and 79 S/cm, respectively. It is worth mentioning that a higher MXene dispersion concentration accompanied by a larger lateral size results in higher electrical conductivity, while deteriorating the strain to failure. Figure 22b–f presents details about MXene–PU nanoyarns that are intended to serve as a piezoresistive sensor with an electrical conductivity of 25 S/cm. The fabrication method uses an MXene bath with a concentration of 4.5 mg/mL, resulting in 19 wt% MXene intake, as determined by TGA. The GF is defined as $\frac{R-R_{0}}{R_{0}.\varepsilon }$ and reported to be 17 within the tensile strain range of 20–50%. Figure 22f shows the piezoresistive response of the MXene–PU nanoyarn under bending deformation ranging from 40° to 65°. Uzun et al. [279] developed a washable, electroactive cellulose yarn (cotton, bamboo, and linen) dip-coated with a Ti_3_C_2_T_x_ MXene aqueous dispersion with a concentration of 25.30 mg/mL, and 77 wt% MXene loading (2.2 mg/cm) endows the yarn a high conductivity of 440 S/cm, which makes it suitable for wearable electronic applications. Knittability was also demonstrated using an industrial knitting machine with the interlock knitting method. According to Figure 23a, the coating process was optimized with a wise strategy to incorporate the highest MXene loading. Figure 23b demonstrates that dip-coating with small-sized MXene flakes effectively covers internal surfaces, while large flakes coat the entire yarn. The washability of the coated yarn was examined after 45 washing cycles at temperatures ranging from 40 °C to 65 °C, while only a minimal increase in electrical resistance was observed. Based on the information in Figure 23c, a capacitive pressure sensor structure was proposed using the knittable coated yarn as an electrode. The observations include a specific capacitance of 759.5 mF cm^−2^ at 2 mV s^−1^, a GF of 6.02, and stable performance over 2000 cycles.

**Figure 24:**
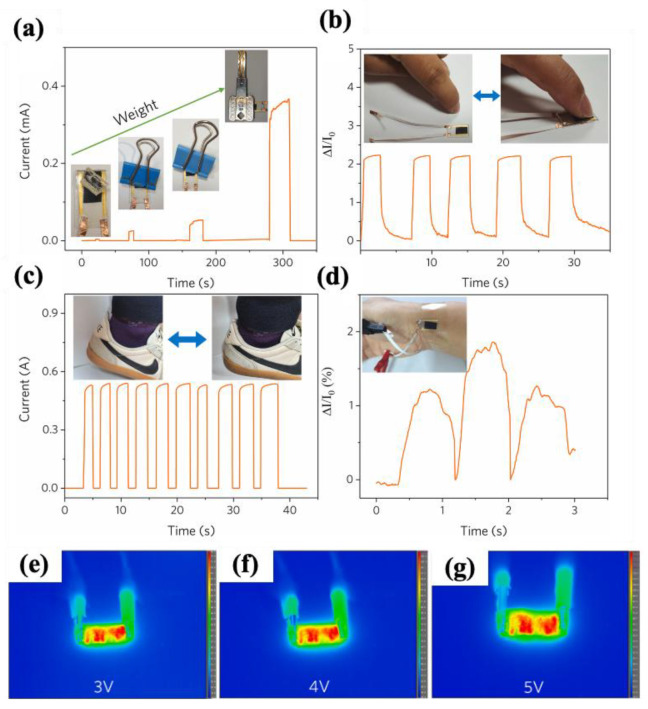
Pressure-sensing functionality of MXene-coated cellulose nonwoven fabric: (**a**) response to different weights, (**b**) finger pressing, (**c**) walking, and (**d**) beating pulse; electrothermal performance under different voltages: (**e**) 3 V, (**f**) 4 V, and (**g**) 5 V [280]. Copyright 2022, Elsevier.

Zhang et al. [280] proposed a breathable MXene-coated cellulose nonwoven fabric for EMI shielding, piezoresistive pressure sensing, and Joule heating functionalities. Simple immersion of the fabric into aqueous MXene dispersion (3 mg/mL) up to nine times, followed by pad-drying, results in a bark-like structure of MXene nanosheets aligned to the surface. The pressure piezoresistive sensor demonstrated a sensitivity of 28.72 kPa^−1^ in a sensing range within 0–17.4 kPa and exhibited durability for more than 2000 cycles. The fast response time of 0.5 s and recovery time of 20 ms are advantages for the real-time monitoring of human activities, like finger pressing, walking, and beating pulse (Figure 24a–d). The EMI-shielding effectiveness of the sample coated and padded nine times was recorded as 35.2 dB across the X-band (8.2 to 12.4 GHz), through reflection (due to the high conductivity) and absorption (attributed to the interlayer spacing and bark-like structure). The Joule heating performance of this fabric is shown in Figure 24e–g under different voltages of 3, 4, and 5 V, with the highest temperature recorded at 146.7 °C for 5 V, while showcasing its capability for personal heating devices.

**Figure 25:**
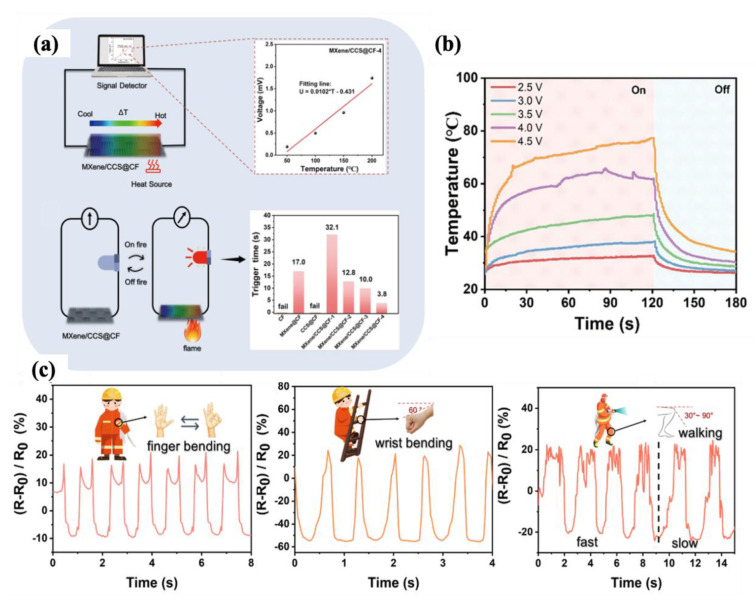
MXene–CCS@CF as (**a**) fire warning (temperature) sensor, (**b**) Joule heating element, and (**c**) piezoresistive motion sensor in different motion modes of finger bending, wrist bending, and walking [281]. Copyright 2021, American Chemical Society.

Wang et al. [281] developed MXene–carboxymethyl chitosan-coated cotton fabric (MXene–CCS@CF) for multifunctional applications, including fire warning, flame retardancy, temperature sensing, and human motion sensing. As shown in Figure 25a, a fire alarm was activated via the thermoelectric properties of MXene, where a temperature difference resulted in a voltage variation for triggering a rapid alarm without an external power source in 3.8 s (compared to traditional alarms with 100 s response time). Flame retardancy of this fabric was achieved by the synergistic carbonization of MXene and CCS, forming a compact char layer with titanium oxide particles. This reduced the peak heat release rate and limited the oxygen index (LOI) by 66.9% and 45.5%, respectively. Joule heating of the fabric showed a surface temperature of ~75 °C at 4.5 V (Figure 25b). The piezoresistive motion sensing for different motion modes is shown in Figure 25c, illustrating stable resistance changes under varying strain (1.5–4.5%) and effectively distinguishing between different motions.

**Figure 26:**
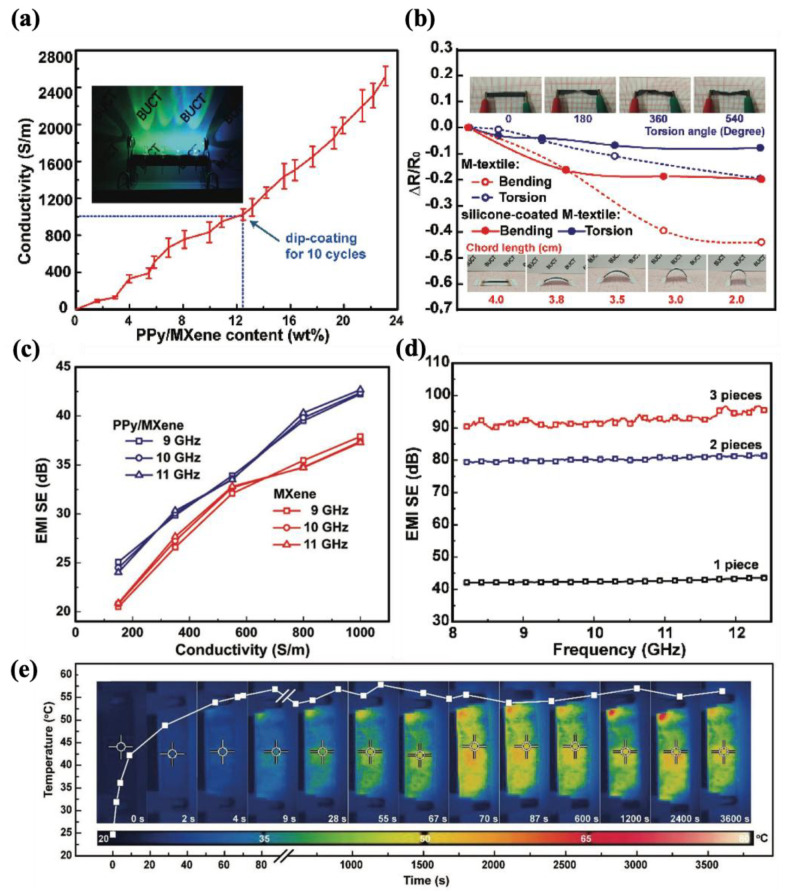
Details of PPy–MXene-coated PET textile: (**a**) electrical conductivity versus nanomaterial’s loading (wt%), (**b**) deformation sensing for non-coated and silicone-coated samples in two torsion and bending modes, (**c**) EMI-shielding effectiveness versus electrical conductivity across different frequencies, (**d**) EMI-shielding effectiveness versus frequency for varying numbers of stacked pieces, and (**e**) Joule heating performance and temperature profile versus time at 3V [282]. Copyright 2018, Wiley-VCH.

**Figure 27:**
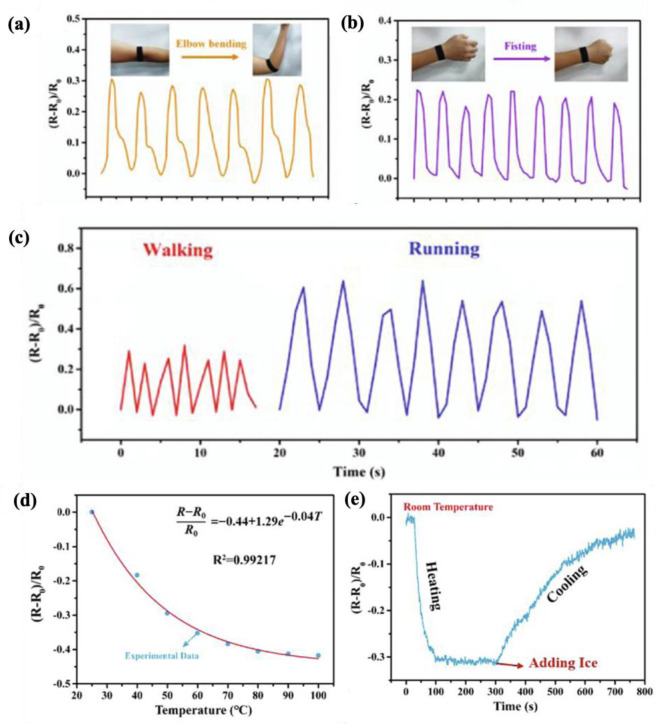
Details of the superhydrophobic breathable multifunctional textile: strain sensing for different movement modes of (**a**) elbow bending, (**b**) fisting, (**c**) walking and running, (**d**) electrical resistance changes versus temperature, and (**e**) versus time in heating-cooling cycle [283]. Copyright 2021, Elsevier.

Wang et al. [282] fabricated a PPy-modified MXene-coated PET-based textile for strain sensing, Joule heating, EMI shielding, and water resistance applications. Pyrrole was polymerized in situ on the surface of Ti_3_C_2_T_x_ MXene to enhance the electrical conductivity and stability. The PET textile was dip-coated multiple times in PPy–MXene ink (6 mg/mL) and subsequently coated with silicone to impart hydrophobicity. Figure 26a represents the relationship between the number of dip-coating cycles and the resultant weight percent of MXene and electrical conductivity. Ten dip-coating cycles resulted in an almost 13 wt% nanomaterial loading and an electrical conductivity of 1000 S/m. The strain-sensing capability of the smart coated textile is represented in Figure 26b in two torsion and bending modes from 0° to 540° torsion and 4 cm to 2 cm chord length, respectively. Though the non-coated textile showed a decent sensitivity, the silicone coating demolished the sensitivity and led to smaller deformation saturation. Moreover, good durability was recorded, with a marginal resistance increase from 32 Ω to 61 Ω over 1000 cycles. Figure 26c,d illustrates the EMI-shielding effectiveness of the coated textile versus the frequency for different electrical conductivities and varying numbers of stacked coated pieces across the X-band. For one piece of textile coated ten times, an EMI-shielding effectiveness of 42 dB was recorded, while it significantly increased to 90 dB when three pieces were stacked. The best electrothermal performance of the coated textile was observed at 4 V, for which a surface temperature of 79 °C was recorded. Figure 26e shows a stable temperature of 57 °C at 3 V, which was maintained for over 3600 s. Silicone coating resulted in an increase in the contact angle from 57° to 126°, ensuring long-term use in high humidity environments. Luo et al. [283] developed a superhydrophobic breathable multifunctional textile for strain and thermal sensing, as well as Joule heating. An elastic polypropylene textile’s surface was modified using dip-coating in PDA to enhance adhesion, followed by dipping into a Ti_3_C_2_T_x_ MXene colloidal solution (5 mg/mL) and coating with polydimethylsiloxane (PDMS) to provide water resistance and durability. The strain-sensing performance of the eight-times-coated textile, with an electrical conductivity of 120 S/m, was reported to have a GF of 18 for strains up to 45% with durability for over 500 cycles. Figure 27a–c shows the strain-sensing capability for detecting human motion monitoring in different modes of elbow bending, fisting, walking, and running. The temperature sensing of the multifunctional fabric was assessed by measuring the temperature coefficient resistance (TCR), which was −1.8 %/°C, within an operating range of 25 °C to 100 °C. It is worth mentioning that temperature sensing was diagnosed by an exponential negative temperature coefficient (NTC) as a result of the destruction of the conductive network induced by matrix thermal expansion, according to Figure 27d,e. Joule heating behavior demonstrated a surface temperature of 89.4 °C at 14 V. The coated fabric’s super hydrophobicity and breathability assessment resulted in a contact angle of 151.4° and vapor transmission rate of 0.49 kg/m^2^ h, respectively.

**Figure 28:**
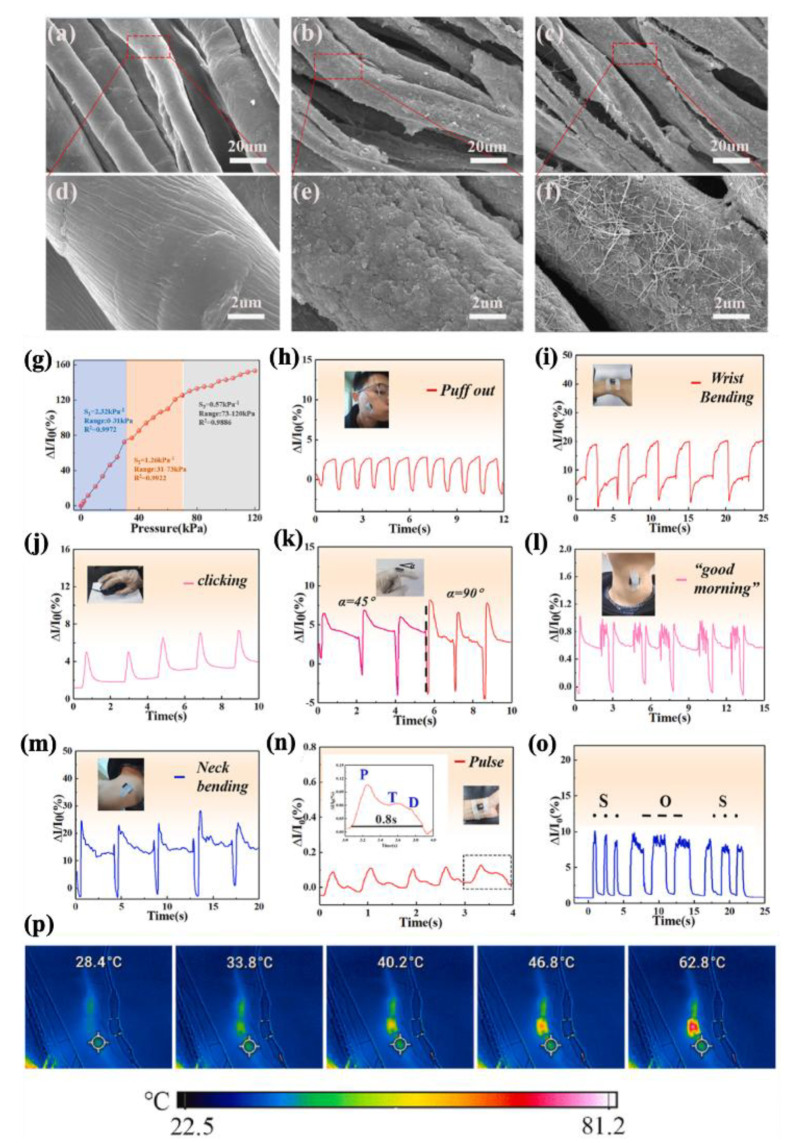
SEM images of MXene–Ag NW-coated cotton fabric: (**a**,**d**) pristine fabric, (**b**,**e**) MXene-coated fabric, and (**c**,**f**) Ag NW-coated MXene@fabric, (**g**–**o**) strain-sensing performance of different human movements and vocal signals, and (**p**) electrothermal performance of Ag NWs-coated MXene@fabric for 15-times coating at 4.5 V [284]. Copyright 2023, Elsevier.

**Figure 29:**
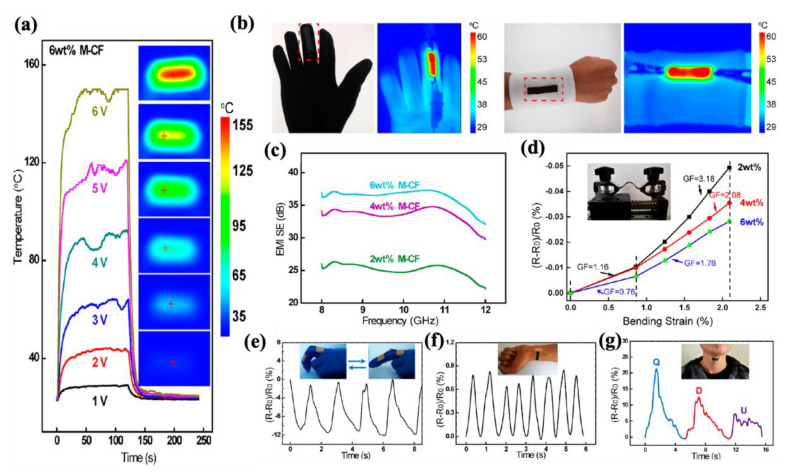
Details of MXene-decorated cotton fabric: (**a**) Joule heating performance of the fabric with 6 wt% MXene loading at different voltages, (**b**) application of the Joule heating element for personal heaters, (**c**) EMI-shielding effectiveness for different MXene loadings across the X-band, (**d**) strain sensitivity for different MXene loadings, and (**e**–**g**) strain-sensing signals for various movements and vocal signals of different letters [285]. Copyright 2020, American Chemical Society.

**Figure 30:**
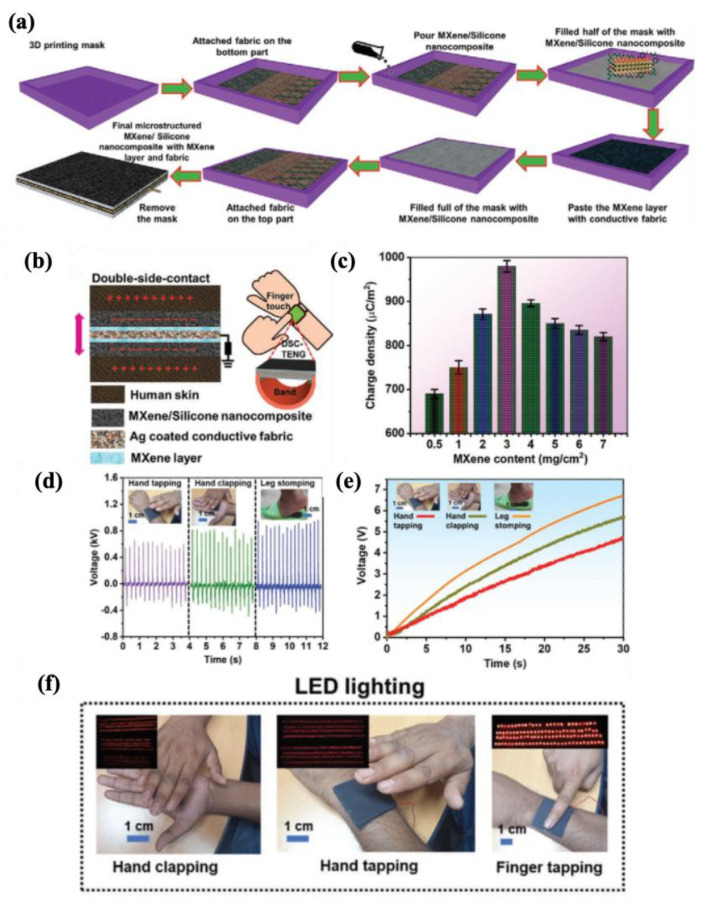
(**a**) The fabrication process for fabric-assisted micropatterning, (**b**) double-sided contact-based TENG, (**c**) charge density of the TENG at different MXene concentrations, (**d**,**e**) sensing application and voltage generation for different loading modes, and (**f**) real application of the TENG for lighting LEDs with varying intensities [286]. Copyright 2021, Wiley-VCH.

**Figure 31:**
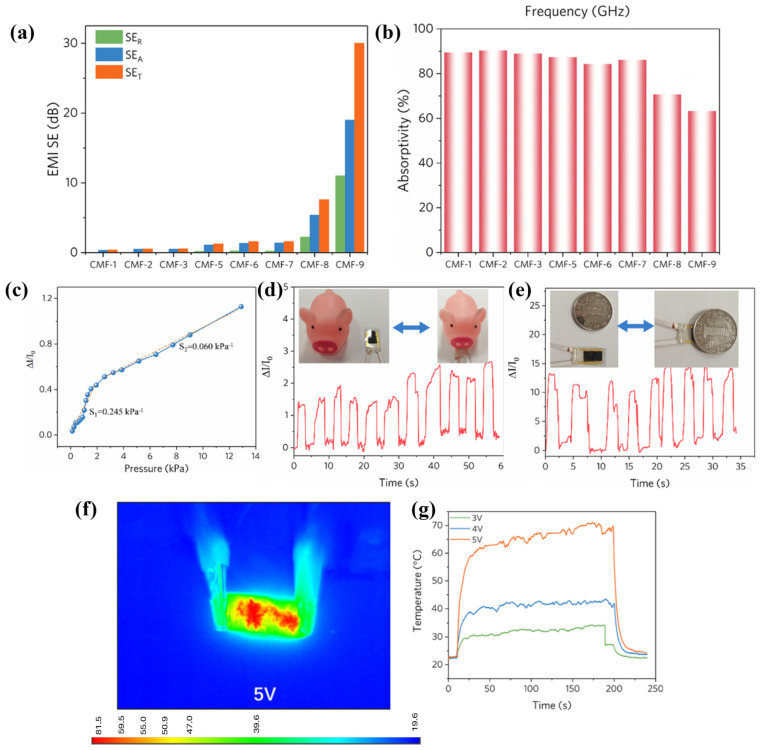
(**a**,**b**) EMI-shielding effectiveness and absorptivity of the bark-shaped CNT–MXene textile over different coating cycles, (**c**–**e**) pressure-sensing performance as a function of pressure and time for different measured objects, and (**f**,**g**) electrothermal performance of the fabric coated nine times at different voltages [287]. Copyright 2021, Elsevier.

**Figure 32:**
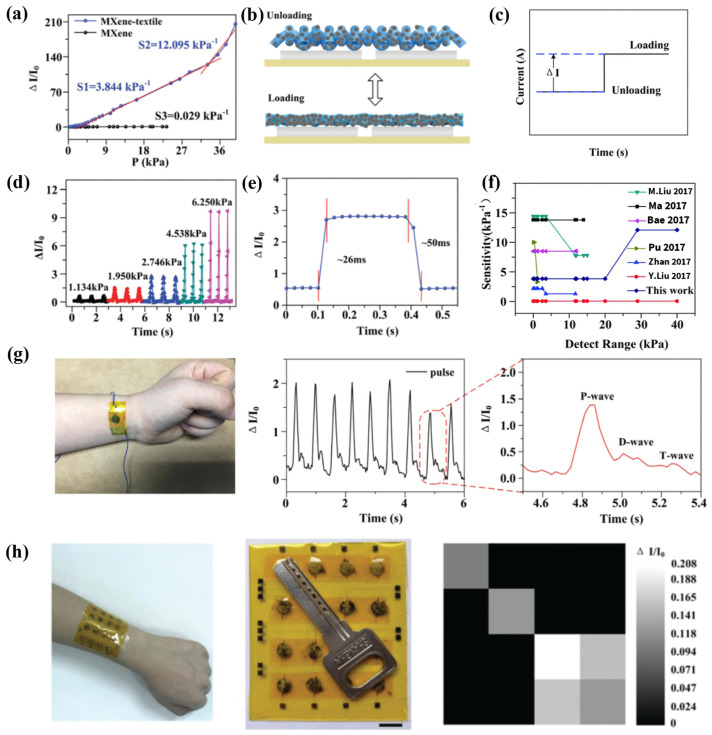
Details of the MXene textile-based flexible and wearable pressure sensor: (**a**) sensitivity over pressure, (**b**,**c**) sensing mechanism, (**d**) pressure sensing for different applied pressures, (**e**) response and recovery times, (**f**) comparison of the sensitivity across the sensing range in this work, [4,19,22,25,44,45] (**g**) heart pulse-sensing performance, and (**h**) pressure-mapping setup [288]. Copyright 2019, Royal Society of Chemistry.

Wang et al. [284] reported MXene–Ag NW-coated cotton fabric for pressure sensing, EMI shielding, and Joule heating multifunctionalities. In this research, a cotton textile (Figure 28a,d) was treated with poly (diallyl dimethyl ammonium chloride) (PDAC) to enhance Ti_3_C_2_T_x_ MXene adhesion. MXene was then applied to the surface through immersion (Figure 28b,e), followed by spray coating with Ag NWs (Figure 28c,f). PDMS was used to encapsulate the coated fabric to improve durability and water resistance. Figure 28g represents the pressure sensor’s ability, with a GF of 2.32 kPa^−1^ across a wide pressure range of 2 to 120 kPa, demonstrating durable performance over 2000 cycles. Figure 28h–o shows the pressure-sensing signals for different human movements, such as puff off, wrist bending, clicking, finger bending, neck bending, heart pulse, and vocal signals like “good morning” and “SOS”. Comparing different signals, well-distinguishability with good repeatability can be implicated. EMI shielding of the 15-times-coated fabric showed an effectiveness of 40 dB, dominated by an absorption mechanism due to the porous nature of the coating combined with high electrical conductivity. Joule heating performance of the same sample is demonstrated in Figure 28p using an infrared camera at 4.5 V, showing a temperature of 63 °C with stable performance over multiple cycles. Zhang et al. [285] fabricated an MXene-decorated cotton fabric by a simple spray coating and drying for 12, 25, and 35 cycles, resulting in MXene loading of 2, 4, and 6 wt%. This fabric showed multifunctionality, including pressure sensing, EMI shielding, and Joule heating. Figure 29a,b demonstrates the electrothermal performance of the coated fabric with 6 wt% MXene loading, which shows surface temperatures ranging from 29 °C at 1 V to 150 °C at 6 V. As shown in Figure 29c, EMI-shielding effectiveness of the decorated fabric increased with increasing MXene loading, with 36 dB effectiveness recorded for 6 wt% MXene loading across the X-band. Pressure sensitivity of the MXene-decorated fabric with 2 wt% MXene loading was found to be the highest, measured at 1.16 and 3.18 for low and high strain rates, respectively (Figure 29d). According to Figure 29e–g, the sensor was capable of detecting various motions and vocal signals of different letters with decent distinguishability. Salauddin et al. [286] developed a fabric-assisted MXene–silicone nanocomposite for motion detection and triboelectric nanogenerators (TENG), which is suitable for self-powered wearable electronics. Figure 30a represents the fabric-assisted micropatterning procedure that is designed for fabricating a double-sided contact-based TENG (Figure 30b). In this design, the highly negative MXene–silicon nanocomposite surfaces functioned as charge-generating layers, while MXene layers and conductive fabrics served as charge-trapping layers and electrodes, respectively. Utilizing the voltage that was generated by movement or force-related stimuli was shown to be particularly useful for self-powered wearable electronics and sensors. The specification of the TENG can be summarized as 1.47 kV at 6 N and 4 Hz. The current and charge density for the optimum MXene concentration of 3 mg/cm^2^ are 200 mA/m^2^ and 980 mC/m^2^ (Figure 30c), respectively. Figure 30d,e shows the sensing capability of the fabricated micro-patterned fabric, which operates based on voltage generation under different force modes. A practical application of lighting LEDs with different intensities using varying touching modes is illustrated in Figure 30f. Zhang et al. [287] developed a multifunctional bark-shaped CNT–MXene cellulose nonwoven fabric for pressure sensing, EMI shielding, and Joule heating applications. The fabric was fabricated by dipping it in a CNT aqueous dispersion, followed by a roll-to-roll process to remove the excess water, and then, repeating the same procedure with MXene dispersion for one to nine cycles. Figure 31a,b represents the EMI-shielding performance of the coated fabric across different cycles. Accordingly, increasing the coating cycles from eight to nine resulted in a significant jump in the EMI-shielding efficiency, reaching 30 dB across the X-band, primarily dominated by absorption (63.3%) thanks to multiple reflection. Pressure-sensing performance is summarized in Figure 31c–e, showing a GF of 0.245 kPa^−1^ for the pressure range from 0.128 to 1.9 kPa. Moreover, the pressure sensor could effectively distinguish between objects of different weights while maintaining a stable output over 5000 cycles. According to Figure 31f,g, the Joule heating application of the multifunctional fabric was evaluated at different voltages ranging from 3 V to 5 V. The surface temperature reached 70.9 °C at 5 V, which surpasses the results for PEDOT–MXene@cotton at 6 V by 44.6 °C. Li et al. [288] fabricated a wearable and flexible MXene–textile (cotton fabric) pressure sensor by employing a simple dip-coating in MXene dispersion (400 mg in 3 mL of ethanol), followed by drying. The coated fabric was then sandwiched between interdigitated electrodes and encapsulated with polyimide (PI). Figure 32a–d represents the pressure-sensing property and its mechanism, where applying pressure enhanced the conductive network connections, leading to an increase in the electrical current, alongside well-distinguishability, between the different applied pressure intensities. According to Figure 32f, the GF was 12.095 kPa^−1^ for pressures between 29 and 40 kPa and 3.844 kPa^−1^ for pressures below 29 kPa, outperforming other counterparts with a decent sensitivity and a wide sensing range. The response and recovery times are 26 ms and 50 ms, with a stable performance for 5600 cycles (Figure 32e). Figure 32g shows the performance of the pressure sensor in detecting heart pulses with a good distinguishability between different waves. As shown in Figure 32h, this pressure-sensitive fabric can be patterned for pressure-mapping applications with the wearability property.

**Figure 33:**
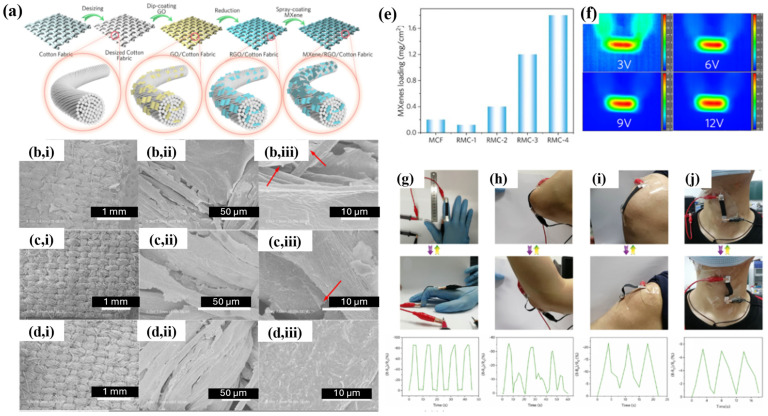
Details of (RGO)–MXene-coated cotton fabric: (**a**) fabrication method, SEM images of (**b**,**i**–**iii**) RGO-coated, (**c**,**i**–**iii**) MXene-coated, and (**d**,**i**–**iii**) RGO–MXene fabrics, (**e**) MXene loading (mg/cm^2^) for different MXene spray coating, (**f**) electrothermal performance with IR camera mapping at different voltages, and (**g–j**) strain-sensing performance for different joint movements [289]. Copyright 2021, Elsevier.

**Figure 34:**
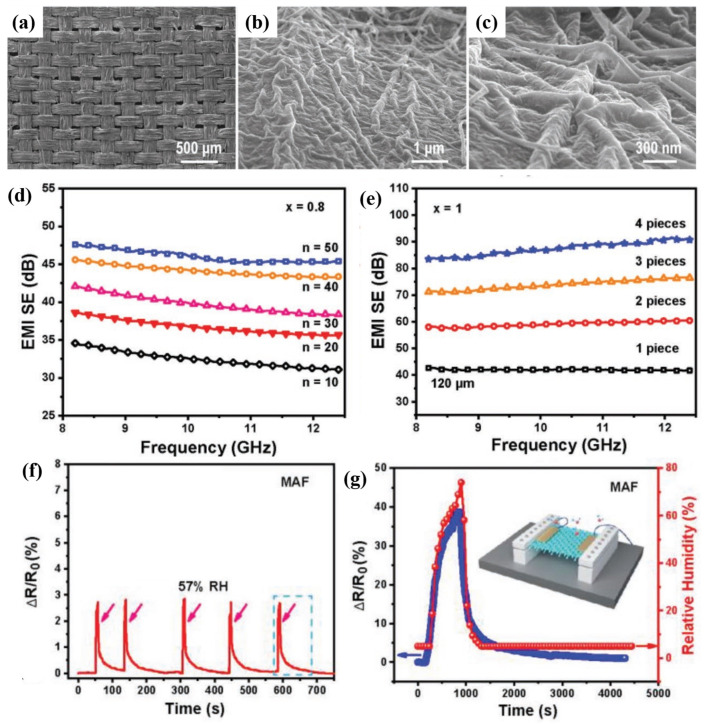
Details of silk fiber alternately coated with Ti_3_C_2_T_x_ MXene nanosheets and Ag NWs, (**a**–**c**) SEM images of leaf-like surface morphology at different magnifications, (**d**) EMI-shielding effectiveness as a function of coating cycles for Ag NWs with a concentration of 0.8 mg/mL, (**e**) EMI-shielding effectiveness as a function of layers number (thickness) form 1 to 4 layers (120 μm to 480 μm) for samples coated for 10 cycles using Ag NWs with a concentration of 1 mg/mL, and (**f**,**g**) humidity sensing performance versus time and humidity percentage. [290]. Copyright 2019, Wiley-VCH.

Zheng et al. [289] fabricated a multifunctional reduced graphene oxide (RGO)– Ti_3_C_2_T_x_ MXene-coated cotton fabric for applications in energy storage, human motion sensing, Joule heating, and EMI shielding. Cotton fabric provided flexibility and breathability, while RGO enhanced the electrical conductivity and surface area. Figure 33a illustrates the fabrication process of the coated fabric, starting with desizing the fabric, followed by dispersion coating with GO, reducing the coated layer to produce RGO, and finally spray-coating with an MXene dispersion. Figure 33b–d shows SEM images of RGO-coated fabric, MXene-coated fabric, and RGO–MXene-coated fabric, respectively. According to Figure 33e, increasing the number of spray-coating cycles increased the concentration of MXene on the surface, with the biggest jump observed between cycles 3 and 4 by an increase of 0.6 mg/cm^2^. The energy storage parameters for the three-times-coated fabric were summarized as a gravimetric specific capacitance of 683.3 F g^−1^ and an areal specific capacitance of 298 mF cm^−2^. The EMI-shielding performance of the coated fabric was reported as 29.04 dB across the X-band, dominated by absorption due to a porous fabric structure. The Joule heating performance of the fabric at different voltages is shown in Figure 33f, with a surface temperature of 66.7 °C recorded at 12 V. It is worth mentioning that the Joule heating efficiency is ΔT= 36 °C for four cycles of spray-coating of MXene. The strain-sensing performance is elaborated in Figure 33g–j, which implies well-distinguishability between different signals, such as the bending of different joints. The sensor exhibited a negative GF of −7.67, which can be attributed to more closure between the fabric yarns. The most relative resistance change was recorded for finger joint bending up to −85.6%. Liu et al. [290] developed a multifunctional flexible silk textile with a biomimetic leaf-like morphology for applications in humidity sensing, EMI shielding, and hydrophobicity. The silk fiber is first treated with oxygen plasma and coated with polyetherimide to enhance the adhesion of conductive fillers. Subsequently, the fiber was alternatively coated with Ti_3_C_2_T_x_ MXene nanosheets and Ag NWs using vacuum-assisted layer-by-layer assembly for various times. The hydrophobicity of the samples was achieved by the mean of two different methods: first, by aging under ambient conduction to alter the surface characteristics, and second, by treating the textile with hydrophobic fluorocarbon agents, such as perfluorooctyltriethoxysilane (POTS), to achieve super-hydrophobicity. Figure 34a–c illustrates the surface morphology of the coated textile at different magnifications. A leaf-like nanostructure can be observed, with MXene sheets serving as the lamina and Ag NWs as the veins (conductive skeleton). Figure 34d,e shows the EMI-shielding effectiveness as a function of the coating cycles for an Ag NW concentration of 0.8 mg/mL and as a function of the number of layers for an Ag NW concentration of 1 mg/mL, respectively. The EMI-shielding effectiveness of a single layer with a thickness of 120 μm, which was coated 10 times with an Ag NWs ink concentration of 1 mg/mL, was reported as 42 dB, exceeding 90 dB for four layers. The aging process induced hydrophobicity by the annihilation of hydrophilic terminal groups but simultaneously led to a deterioration in the EMI-shielding effectiveness. The humidity-sensing performance of the coated fabric is summarized in Figure 34f,g, showing a response time of 5 s and a recovery time of 80 s at 57% room humidity. The humidity-sensing mechanism was attributed to the increasing trend in electrical resistance by increasing the humidity that is caused by water vapor interacting with the MXene interlayer spaces. Table 8 summarizes the findings and highlights the studies on fabric-based multifunctional MXene materials for sensing and Joule heating applications.

#### 4.2.2. Hydrogel-Based Multifunctional Sensors and Heaters

**Figure 35:**
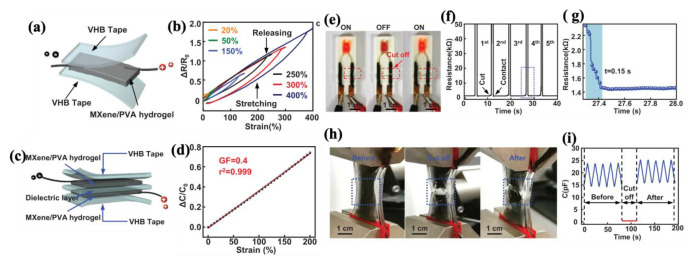
MXene–PVA hydrogel: (**a**,**c**) piezoresistive and capacitive MXene–PVA hydrogel sensors, respectively, (**b**) relative resistance change (piezoresistive performance) versus strain, (**d**) highly linear relative capacitance change versus strain, and (**e**–**i**) self-healing capability of the multifunctional hydrogel with a fast healing time and performance recovery after healing [291]. Copyright 2019, Wiley-VCH.

**Figure 36:**
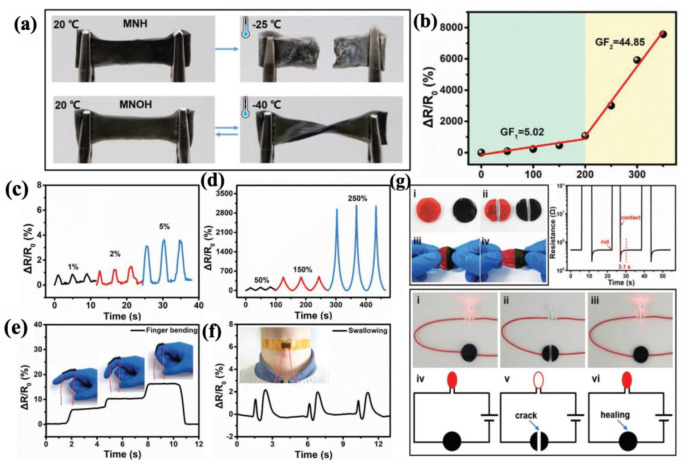
Details of MNOH: (**a**) anti-freezing property, (**b**) relative resistance change versus strain, (**c**,**d**) sensing accuracy over time from small to high strains, (**e**,**f**) strain-sensing performance during finger bending and swallowing, and (**g**) self-healing performance [292]. Copyright 2019, Wiley-VCH.

Another extensively studied class of MXene-based multifunctional materials is hydrogels, known for their extraordinary flexibility and self-healing potential. Zhang et al. [291] developed a highly stretchable and healable Ti_3_C_2_T_x_ MXene–PVA hydrogel for capacitive and piezoresistive sensing, offering excellent linearity for applications in electronic skin (E-skin), personalized medicine, AI devices, and soft robotics. For fully mimicking human skin, E-skin must be highly stretchable and indeed healable. This hydrogel showed a strain at break of 1200% and a super quick self-healing of 0.15 s. Capacitive strain sensors have been reported to detect delicate movements with low hysteresis, like eye blinking and pulse detection. Figure 35a illustrates the piezoresistive sensor’s structure, while Figure 35b shows the relative resistance change under different strains, by which significant hysteresis is indicated. On the other hand, the capacitive-sensing performance, shown in Figure 35c, demonstrates great linearity in a hysteresis-free mode, with a GF of 0.4 and excellent linearity up to 200% strain, as seen in Figure 35d. Additionally, only a 5.8% reduction in capacitance retention was recorded after 10,000 cycles. The self-healing capability of the multifunctional hydrogel, as shown in Figure 35e–i, indicates very fast healing and excellent performance recovery after healing in both the piezoresistive and capacitive sensors. The borax crosslinker increased the self-healing efficiency of the hydrogel and formed a covalent bond with MXene sheets. The capacitive sensor successfully detected delicate movements, such as induced epidermal movements, water dripping, and finger bending. Liao et al. [292] developed an MXene nanocomposite organo-hydrogel (MNOH) for strain sensing, self-healing, and anti-freezing properties. Ti_3_C_2_T_x_ MXene was mixed with polyacrylamide (PAAM) and PVA to form a hydrogel blend, along with 4 wt% borax for dynamic crosslinking. Polyethylene glycol was added as a water replacement to impart anti-freezing properties. Figure 36a represents the anti-freezing property of the MNOH, which maintains its stretchability at −40 °C. According to Figure 36b, the strain sensitivity of this hydrogel was 5.02 for 0–200% and 44.85 for 200–350% strain, respectively. It is worth noting that the strain detection limit was 0.1% (Figure 36c), which enables the sensor to detect subtle movements such as biological signals (Figure 36e,f). Moreover, a great distinguishability between the different applied strain intensities and the extraordinary stretchability, ranging from 50 to 250%, is featured (Figure 36d). The self-healing property of the organo-hydrogel was evidenced by retaining 85% of its tensile strength within 12 h, while the electrical resistance was fully restored within 3.1 s, as shown in Figure 36g.

**Figure 37:**
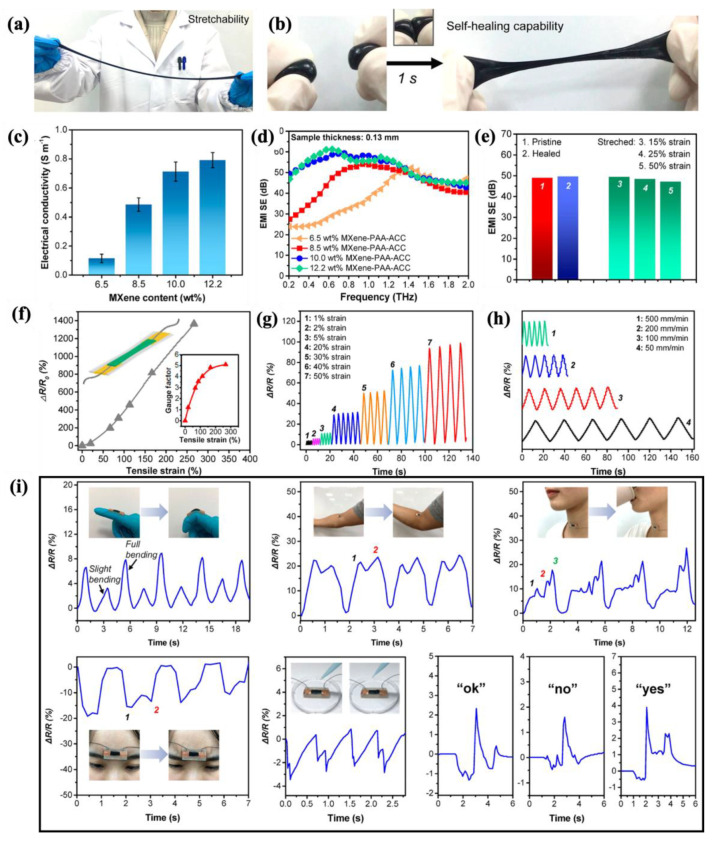
Multifunctional Ti_3_C_2_T_x_ MXene composite hydrogel: (**a**) stretchability, (**b**) self-healing, (**c**) electrical conductivity versus MXene weight percent, (**d**,**e**) EMI-shielding effectiveness, (**f**–**h**) strain-sensing performance, and (**i**) movement detection for various subtle movements, such as finger and elbow bending, swallowing, forehead movements, water droplet falling, and speech recognition [293]. Copyright 2021, American Chemical Society.

**Figure 38:**
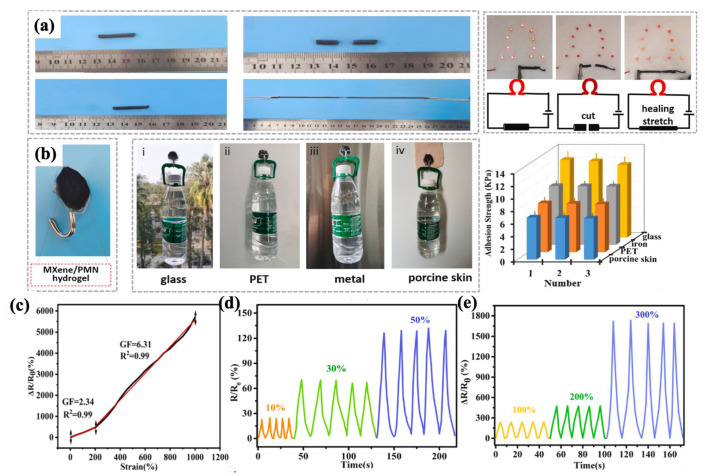
Ti_3_C_2_T_x_ MXene–polyampholytes hydrogel (**a**) self-healing property, (**b**) self-adhesion on different substrates, (**c**) relative resistance change versus strain, and (**d,e**) relative resistance change for different strain ranges [56]. Copyright 2022, Elsevier.

**Figure 39:**
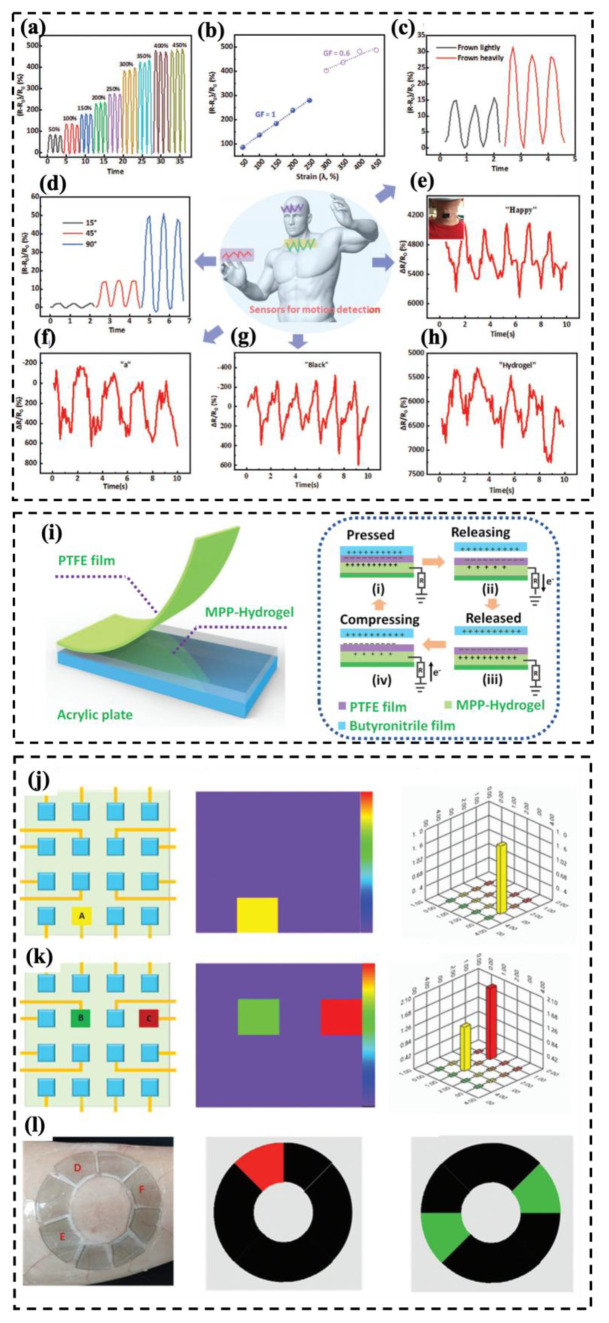
Information on PAAM/PVA double-network coated hydrogel (**a**–**h**) strain-sensing functionality, (**i**) structure and working mechanism of the developed TENG, and (**j**–**l**) the performance of the developed E-skin and pressure-mapping capability (capital letters inside the figure (A–F) specify the pressed array(s) with different forces) [295]. Copyright 2022, Wiley-VCH.

Zhu et al. [293] fabricated a multifunctional Ti_3_C_2_T_x_ MXene composite hydrogel based on PAA with excellent shape adaptability and recyclability for applications in strain sensing, absorption-dominated EMI shielding, and self-healing. Figure 37a demonstrates the hydrogel’s extraordinary stretchability and rapid self-healing, with efficiency achieved in seconds and electrical resistance and tensile toughness restored within 10 min, regarding Figure 37b. Electrical conductivity versus MXene weight percent is summarized in Figure 37c, which indicates a significant increase in the range of 6.5 to 8.5 wt%. The EMI-shielding effectiveness for a 0.13 mm-thick sample at different MXene contents across the terahertz frequency is presented in Figure 37d,e. The shielding effectiveness for samples with 10–12.2 wt% MXene remained nearly identical, while the sample with 8.5 wt% MXene achieved an effectiveness of 45.3 dB. The dominant shielding mechanism was absorption due to moderate conductivity and a water-rich porous structure. The strain-sensing performance of the hydrogel is reported in Figure 37f–h, showing the GF increasing from zero to five as the strain rises, with high sensitivity starting at a 1% strain. Figure 37i highlights the hydrogel’s ability to detect different movements, such as finger and elbow bending, swallowing, forehead movements, water droplet falling, and speech recognition, which showcases perfect distinguishability for subtle movements in the signals. Liu et al. [294] conducted similar research by developing a Ti_3_C_2_T_x_ MXene–PAAM composite organo-hydrogel with a PEG–water hybrid solvent for strain sensing and freezing tolerance. The addition of PEG provided two key benefits: suppressing water loss and imparting anti-freezing. This prevented ice crystallization by forming hydrogen bonds, enabling the hydrogel to operate in a subzero condition (−20 °C) without cracking or losing flexibility. Moreover, the strain-sensing performance was reported with a GF of 6.31 for large strains, which demonstrates high sensitivity and stable relative resistance change over 500 cycles. Chen et al. [56] fabricated an ultra-stretchable, self-healing, and adhesive Ti_3_C_2_T_x_ MXene–polyampholytes hydrogel, with a wearable epidermal sensor design. The optimal MXene concentration for achieving the best electrical and mechanical properties was determined at 1 wt%. Figure 38a illustrates the self-healing property of the multifunctional hydrogel with excellent post-healing stretchability and electrical conductivity. Figure 38b shows the self-adhesion property of the hydrogel to different substrates, including glass, PET, metal, and porcine skin. The hydrogel exhibited consistent adhesion over three attach–detach cycles, with the adhesion strength decreasing in the order of glass > iron > PET > porcine skin, ranging from approximately 13 to 7 kPa. The details of the strain-sensing performance can be found in Figure 38c,d, with GFs of 2.34 and 6.31 for strain ranges of 0–200% and 200–1000%, respectively. The sensor displayed a perfect sensitivity and an ability to distinguish strains of varying magnitudes, making it appropriate for detecting various human body activities, such as joint bending, blinking, swallowing, and speech recognition. Additionally, its strong adhesion ensured reliable deployment on the skin. Long et al. [295] developed a multifunctional double-network hydrogel for pressure and strain sensing, as well as TENG applications. In this research, 0.4 mL of aqueous Ti_3_C_2_T_x_ MXene dispersion (5 mg/mL) were mixed with 8 g of a PAAM and PVA polymer mixture to make a double-network hydrogel, which was crosslinked using ammonium persulfate. Figure 39a–h represents the strain-sensing performance of the double-network hydrogel. As can be seen, the GFs of 1 and 0.6 were reported for strain ranges of 0–250% and 300–450%, respectively. The monitoring of human activities was successfully demonstrated with good distinguishability for various types of movements, like gestures, facial expressions, and different vocal fold vibrations. Figure 39i illustrates the structure and working mechanism of the TENG. The TENG performance was reported to show an open-circuit voltage of 180 V, a short-circuit current of 10 mA, and a transferred charge of 65 nC, making it suitable for self-powered E-skins and energy harvesting applications. It is worth mentioning that MXene sheets formed an electric double layer with water molecules, by which the charge transfer efficiency improved. Finally, a self-powered pressure sensor array was assembled to serve as an E-skin. Figure 39j–l shows the performance of the developed E-skin, which accurately measured the pressure magnitude, mapped the pressure distribution, and distinguished between the number of tapped areas. Table 9 summarizes studies on hydrogel-based multifunctional MXene materials, highlighting their key findings for sensing and Joule heating applications.

#### 4.2.3. Other Types of Structures Multifunctional Sensors

**Figure 40:**
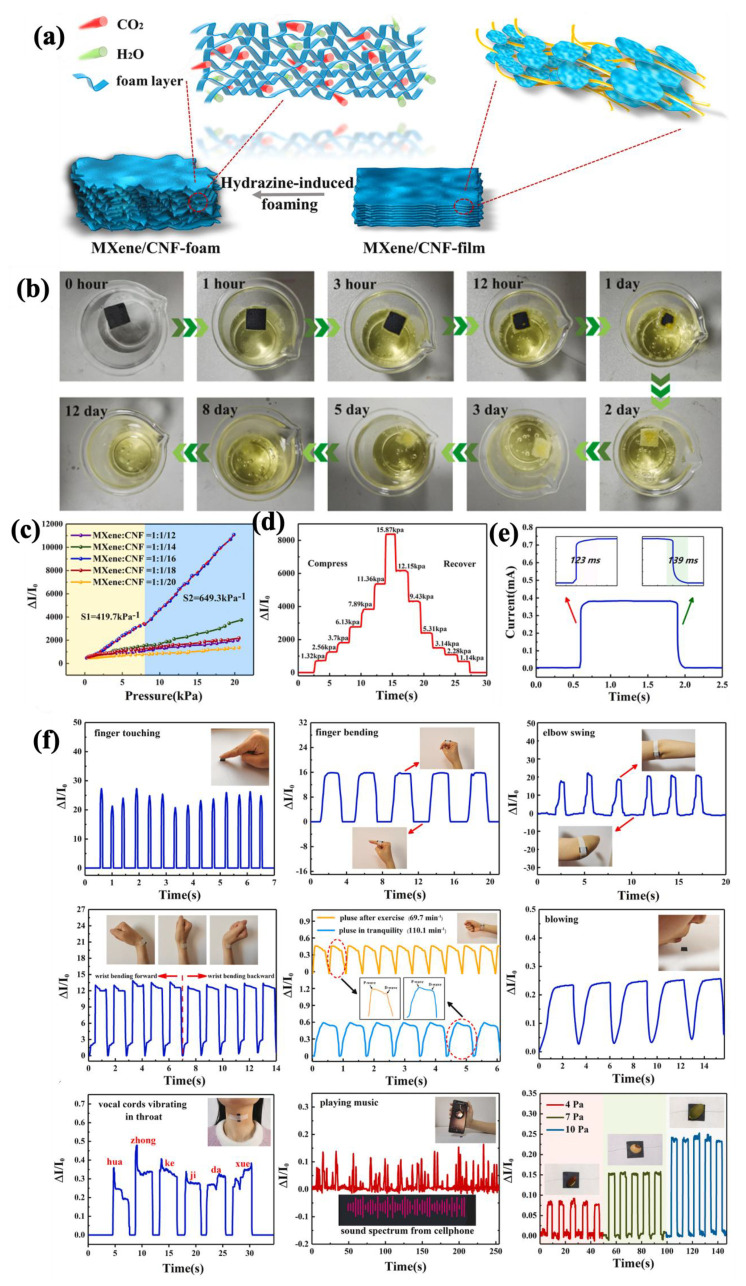
Details of MXene–CNF foam: (**a**) manufacturing process, (**b**) degradation process over 12 days in 1 wt% in H_2_O_2_, (**c**) relative electrical current change over pressure range, (**d**) pressure-sensing for various pressures, (**e**) pressure-sensing response and recovery time, and (**f**) pressure-sensing performance for different human-related activities, such as finger touching and bending, elbow swings, wrist bending, heart pulse in rest and exercise, vocal cord vibration, and playing music [296]. Copyright 2021, Elsevier.

**Figure 41:**
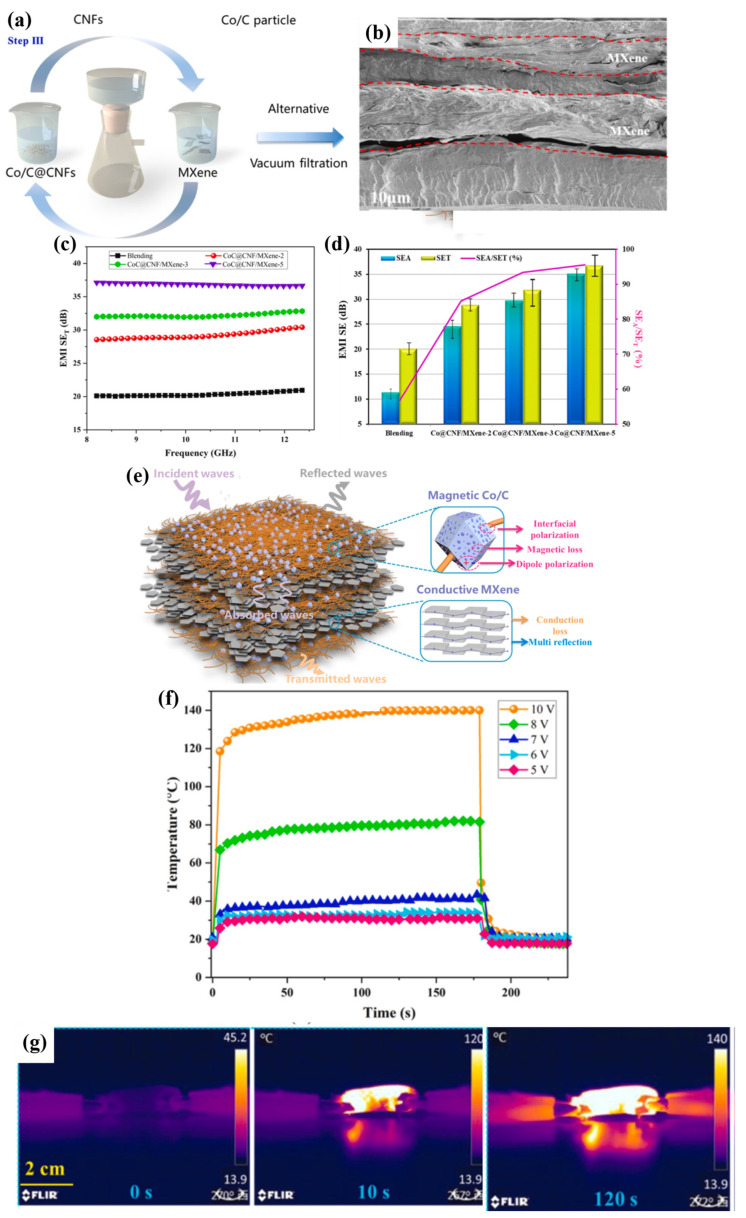
CoC@CNF/Ti_3_C_2_T_x_ MXene hybrid film: (**a**) manufacturing process, (**b**) cross-sectional microstructure, (**c**,**d**) EMI-shielding effectiveness for blending condition and various deposition cycles, (**e**) EMI-shielding mechanism, and (**f**,**g**) thermoelectric performance of the hybrid film at different voltages, and IR camera images of the 5-times-coated sample at 10 V [297]. Copyright 2023, Elsevier.

**Figure 42:**
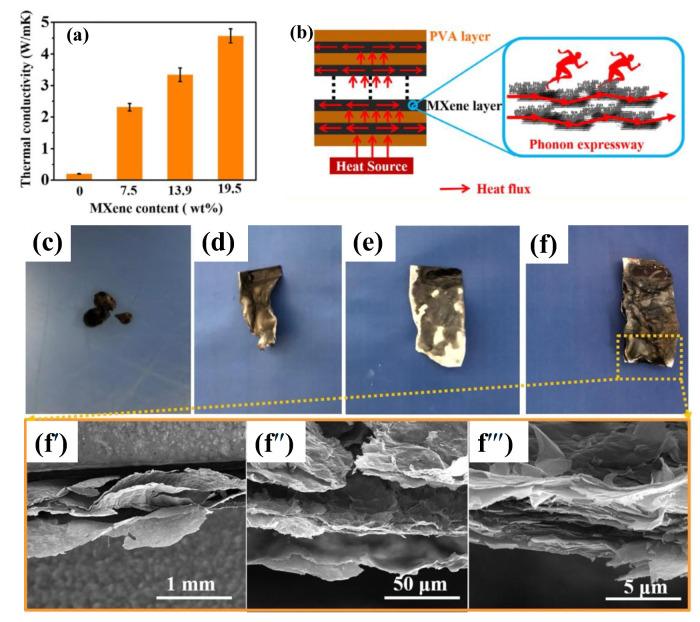
PVA–MXene multilayer: (**a**) thermal conductivity for different MXene contents, (**b**) schematic illustration of the dominant mechanism for improving thermal conductivity, and (**c**–**f**) burnt sample photographs of neat PVA and PVA/MXene multilayers with 7.5, 13.9, and 19.7 wt% MXene, respectively, (**f**′–**f**′′′) SEM images of PVA/MXene multilayer sample with 19.7 wt% MXene under different magnifications [132]. Copyright 2020, Elsevier.

Other kinds of porous structures, such as nanofibers or freeze-dried networks, offer lightweight, high porosity, and customizable microstructures that may be advantageous for enhancing EMI shielding and/or sensitivity. Su et al. [296] developed an MXene–cellulose nanofiber (CNF) foam for pressure sensing and biodegradability. The mixture of CNF and MXene was poured into a vacuum filtration system multiple times to form a multi-layer MXene–CNF film. The vacuum-filtered hybrid freestanding film was then placed into an autoclave filled with hydrazine monohydrate at 90 °C for 10 h to transform the films into a porous structure (Figure 40a). The biodegradability process of the MXene/CNF foam in a low concentration hydrogen peroxide (H_2_O_2_) (1 wt%) medium is shown in Figure 40b, indicating complete degradation within 12 days. The pressure-sensing performance of the MXene/CNF foam is summarized in Figure 40c–f. The GF values of the optimized samples were reported as 419.7 kPa^−1^ and 649.3 kPa^−1,^ corresponding to pressures between 0–8.04 kPa and 8.04–20.55 kPa, respectively. Figure 40d demonstrates a great sensitivity and clear distinguishability between small pressure increments, with low hysteresis during the recovery stage. Response and recovery times of 123 ms and 139 ms, respectively, are shown in Figure 40e. Sensing performance for real human-related movements, such as finger touching and bending, elbow swings, wrist bending, heart pulse in rest and exercise, and blowing, are shown in the first three rows of Figure 40f. The foam exhibited great sensitivity, with dedicated signal patterns for each kind of movement, which enables delicate monitoring. The distinct signal patterns extended the sensing capabilities to vibrational signals, like vocal cord vibration and playing music, with clear pattern distinction. Ran et al. [297] conducted similar research by fabricating a flexible and tough CoC@CNF/Ti_3_C_2_T_x_ MXene multilayer film for EMI shielding and Joule heating applications. The Co/C magnetic nanoparticles, derived from a ZIF-67 metal–organic framework (MOF), enhanced magnetic loss. Figure 41a,b demonstrates the fabrication process of the CoC@CNF/ Ti_3_C_2_T_x_ MXene hybrid. Alternating vacuum filtration of the MXene and CoC@CNF was performed for multiple cycles to obtain a layered structure, as shown in Figure 41b. The optimized electronic results are attributed to the sample with five deposition cycles. The corresponding electrical conductivity and absorption-dominant EMI-shielding effectiveness (95%) (Figure 41c,d) are recorded as 90 S/m and 37.1 dB, respectively. The EMI-shielding mechanism that is visualized in Figure 41e implies the contribution of the magnetic core to interfacial and dipole polarization, which led to the magnetic loss, while the conductive MXene enabled multi-reflection and conduction loss. Electrothermal performance of the sample with five deposition cycles is demonstrated in Figure 41f,g at different applied voltages ranging from 5 to 10 V. The highest recorded temperature was 138 °C at 10 V, with a fast response time and applicable for personal heating applications. The study conducted by Jin et al. [132] on the fabrication of a multilayer PVA–MXene composite film provides another example of this type of macrostructure. The developed multilayer film, consisting of six layers of PVA and five layers of MXene, exhibited the best performance for EMI shielding (44.4 dB), thermal conductivity (4.57 W/mK), and flame retardancy applications. According to Figure 42b, the primary mechanism for enhancing thermal conductivity was attributed to phonon conduction through the structurally ordered MXene sheets. Flame retardancy examination results showed that the neat PVA burned completely with melt-dripping, while the addition of MXene preserved the structural integrity during burning, according to Figure 42c–f. The flame retardancy mechanism was attributed to the formation of TiO_2_ from MXene during burning, which acts as a protective layer against the fire, according to Figure 42f′-f′′′. Nguyen et al. [298] developed a porous graphene foam coated with Fe_3_O_4_@MXene and infused with PDMS for pressure-sensing and EMI-shielding applications. Fe_3_O_4_ nanoparticles were intercalated between MXene sheets as a coating material. The graphene foam was fabricated by CVD deposition of graphene onto a nickel foam, followed by the removal of the nickel base. The final porous and hollow graphene foam was coated with modified MXene and infused with PDMS to provide flexibility and structural integrity. The sample incorporated Fe_3_O_4_@MXene with an 11.53 wt% and exhibited an electrical conductivity of 630 S/cm. Pressure-sensing tests showed a sensitivity of 0.12 kPa^−1^ for pressures ranging from 62.4 to 300 kPa and 0.04 kPa^−1^ for pressures from 300 to 800 kPa. EMI-shielding effectiveness was recorded as 80 dB and 77 dB in the X-band and Ka-band, respectively, dominated by the absorption mechanism (80%). The EMI-shielding mechanism was attributed to the generation of eddy currents in the hollow graphene tubes within the porous structure, along with space interfacial electric polarization from the MXene functional groups and magnetic loss from the Fe_3_O_4_ nanoparticles.

## 5. Future Outlook

Modern industrial growth, urban expansion, and technological advancements have doubled the demand for multifunctional electronic applications. MXenes’ excellent physiochemical properties, such as high conductivity, remarkable electronic properties, layered structure with a high surface area, and hydrophilicity with tunable surface functional groups, have garnered substantial attention since their discovery in 2011. These properties have been further enhanced by incorporating MXene into diverse polymer matrices and/or hybrids, resulting in composites with tailored functionality. Among electronic applications, energy storage, sensory, and Joule heating have emerged as top priorities. Moreover, MXene–polymer composites demonstrate a unique ability to combine high performance with flexibility and lightweight characteristics, which is critical for next-generation electronic devices.

Extensive research has been conducted to advance the design and manufacturing of these nanomaterials. To elaborate, the efforts include synthesizing different types of MXenes tailored to specific applications, optimizing synthesis techniques to enhance their engineered properties, and integrating MXenes with suitable polymer candidates. Looking ahead, immense opportunities exist for expanding multifunctionality, and revolutionizing smart, wearable electronics calls for further research. Key gaps are found in hybridizing MXenes with other nanomaterials, developing advanced composite architectures, and integrating computational modeling with experimental practices. However, several critical challenges must be addressed simultaneously to unlock the full potential of MXene–polymer nanocomposites:

**Synthesis method**: The reliance on fluorine-based reagents in common MXene synthesis methods poses serious risks to human health and the environment. Developing green and sustainable synthesis methods is crucial. Therefore, synthesis techniques must be optimized even for large-scale, cost-effective production while maintaining the optimal size and quality (e.g., minimal defects), as these factors strongly influence properties like electrical conductivity.

**Oxidation and Stability**: MXene’s active surface terminations (e.g., -OH, -F, and =O groups) and large surface area make it prone to rapid oxidation. Thus, MXene materials’ stability and critical properties like conductivity can be degraded substantially. Employing stabilization strategies is essential to extend the devices’ service life and ensure reliable performance in electronic applications.

**Interface engineering**: Achieving strong interfacial interactions between MXene sheets and polymer chains is critical for enhancing composite performance. This becomes particularly challenging when the polymer matrix is nonpolar, requiring advanced surface functionalization techniques.

**Application-specific optimization:** Many electronic applications involve trade-offs between key properties. For instance, in electrostatic capacitors, increasing the dielectric constant often reduces the breakdown strength while enhancing the dielectric loss. Tailoring composites to strike a balance between these properties for specific functions seems essential.

In conclusion, given the novelty of this field, as well as those related to MXene nanomaterials themselves, significant research efforts are still pending in the development of highly efficient, multifunctional MXene–polymer composites for electronic applications. Addressing these challenges will pave the way for innovative materials with transformative potential in electronics, such as energy storage, sensing, and wearable electronics.

## 6. Conclusions

In recent decades, rapid advancements in industry and technology have driven an unprecedented demand for high-performance, lightweight electronic devices. This evolution, coupled with rising energy consumption and environmental concerns, necessitates innovative strategies for developing multifunctional electronic systems. Among various strategies, the incorporation of nanomaterials into polymer matrices as well as hybrid structures, along with a precise microstructure design, offers substantial promise. In particular, the integration of polymers with two-dimensional (2D) nanomaterials, such as MXenes, has shown exceptional potential. These polymer nanocomposites and hybrid materials leverage the light weight, flexibility, and processability of polymers with the superior electrical conductivity, mechanical strength, and tunable surface chemistry of MXenes. This review provides a comprehensive exploration of MXene–polymer nanocomposites and hybrid materials with an emphasis on their multifunctionality in electronic applications. It covers key synthesis methods for MXene production and fundamental properties for electronic applications, both as individual materials and nanofillers. The multifunctional applications discussed include energy storage, sensing, and Joule heating systems, which are pivotal for advancing smart technologies. Finally, the review outlines future research directions, emphasizing the need to address challenges such as large-scale fabrication, long-term stability, and the optimization of multifunctional properties. These efforts are key to realizing the full potential of MXene–polymer nanocomposDeclare ites in next-generation electronic devices.

## Figures and Tables

**Table 1:** A summary of key advantages and disadvantages of synthesis methods considering application-based properties and scalability.

Synthesis Method	Large-Scale Production	
	Application-Based Properties	Scalability
HF direct etching	‑ Appropriate for a wide variety of MXene types ‑ Formation of -F termination groups	‑ Efficient production method ‑ High safety risks ‑ Environmental concerns
Indirect (MILD) etching	‑ Improved quality and stability	‑ Safer and more environmentally friendly alternatives for HF direct etching ‑ Slower production ‑ Less efficient
Electrochemical etching	‑ High quality ‑ Tunable termination groups	‑ Fluorine-free, safer process ‑ High yield ‑ Expensive equipment for large-scale production
Hydrothermal	‑ High quality with reduced defects ‑ Tunable termination groups	‑ Fluorine free ‑ High yield ‑ Low scalability due to harsh conditions

**Table 2:** A summary of effective parameters on electrical conductivity of MXene films.

MXene Type	Synthesis	Delamination	Details/Effective Parameters	Conductivity (S.cm^−1^)	Refs.
Mo_2_CT_x_	HF (50%)	TBAOH	Type of MXene	1300	[30]
Ti_3_C_2_T_x_	MILD	Li^+^	MAX phase: graphite as carbon source	4400	[118]
Ti_3_C_2_T_x_	MILD	Li^+^	MAX phase: TiC as carbon source	3480	[118]
Ti_3_C_2_T_x_	MILD	Li^+^	MAX phase: lampblack as carbon source	1020	[118]
Ti_3_C_2_T_x_	HF (50%)	Li^+^	Excess aluminum in MAX phase	20,000	[119]
Ti_3_C_2_T_x_	MILD	Li^+^	Number of layers: Monolayer	6760	[120]
Ti_3_C_2_T_x_	MILD	Li^+^	Synthesis method: ratio of LiF/HCl	4600	[121]
Ti_3_C_2_T_x_	MILD	Li^+^	Synthesis method: N_2_ atmosphere	14,000	[122]
Ti_3_C_2_T_x_	HF (50%)	DMSO	Delamination treatment	123	[123]
Ti_3_C_2_T_x_	MILD	Li^+^	Removing fluorine-containing surface terminations by annealing treatment	3697	[124]
Ti_3_C_2_T_x_	MILD	Li^+^	Flake size: large size	5000	[125]
Ti_3_C_2_T_x_	MILD	Li^+^	Flake size: small size	1000	[125]

**Table 3:** A summary of studies on electrical properties of MXene/polymer nanocomposites.

Polymer Matrix	MXene/ Loading	Processing Method	Conductivity (S/cm)	Other Functionalities/ Applications	Ref.
Polystyrene (PS)	Ti_3_C_2_T_x_/1.9 vol%	Solution and vacuum filtration	10.81	High EMI SE and mechanical properties/EMI shielding	[135]
Epoxy	Ti_3_C_2_T_x_/15 wt%	Solution casting	1.05	High EMI SE and mechanical properties/EMI shielding	[136]
PEDOT:PSS	Ti_3_C_2_T_x_/87.5 wt%	Solution and vacuum filtration	340.5	High EMI SE and mechanical properties/EMI shielding	[137]
Poly(ethylene oxide) (PEO)/ Succinonitrile (SN)	Ti_3_C_2_T_x_/0.8 wt%	Solution casting	2.17 × 10^−3^	Electrochemical performance, high mechanical properties, and capacity retention/electrolyte	[45]
Copolyimide	Ti_3_C_2_T_x_/5.0 wt%	Solution casting	1.4 × 10^−2^	High electrical, mechanical, and transparency/flexible electronics	[138]
Poly(ethylene oxide) (PEO)	Ti_3_C_2_T_x_/3.6 wt%	Solution blending	2.2 × 10^−7^	High ionic conductivity, rate capability, and stability/electrolyte in batteries	[35]
Polyaniline	Ti_3_C_2_T_x_	In situ polymerization	88.4	High specific capacitance and capacitance retention/ electrode in supercapacitors	[44]
PVDF/ PMMA	Ti_3_C_2_T_x_/15.0 wt%	Solution blending	10^−3^ @ 1 MHz frequency	High energy and power density/dielectric materials in capacitors	[139]
Polyurethane (PU)	Ti_3_C_2_T_x_/23.1 wt%	Wet spinning	22.6	High gauge factor, large sensing strain/strain sensors in health and sports	[140]

**Table 4:** A summary of studies on dielectric properties of MXene–polymer nanocomposites.

Polymer Matrix	Filler(s)/ Loading	Processing Method	$\varepsilon^{\prime }$ $/\varepsilon^{\prime \prime }(\tan\;\delta )$	Applications	Refs.
PVDF/PMMA	MWCNT ^a^/Ti_3_C_2_T_x_ 15 wt%	Solution casting	246/(~0.2) @ 100 Hz	Capacitors	[158]
PVA	Ti_3_C_2_T_x_ @SiO_2_ 2.5/5 wt%	Solution coating	27.2/(0.057) @ 100 Hz	Electronics	[159]
paraffin	Ti_3_C_2_T_x_ 6.67 vol%	Melt mixing	~37.5/~52 @ 2 GHz	Microwave absorption	[160]
P[VDF-TrFE-CFE]	L-Ti_3_C_2_T_x_ 15.3 wt%	Solution casting	139,830/4.1 @ 1 kHz	Electronics, capacitors, and triboelectric generators	[161]
PPy	Ti_3_C_2_T_x_ 0.8 g	In situ polymerization	11.61/6.47 @ 2 GHz	Microwave absorption	[162]
Epoxy	Ti_3_C_2_T_x_ 30 wt%	Solution mixing	~34/(~0.45) @ 2 GHz	Microwave absorption	[163]
PVA	Ti_3_C_2_T_x_ 20 mg/cm^2^	Directional freezing of hydrogel	~21 @ 10 GHz	Electromagnetic shielding	[164]
P(VDF-HFP)	Ti_3_C_2_T_x_ 4 wt%	Solution casting	539/0.06 @ 1 kHz	Energy storage applications	[165]
PMMA	Ti_3_C_2_T_x_ 50 mg	Solution casting	22/6.5 @ 2 GHz	Microwave absorption	[166]
PVA	Ti_3_C_2_T_x_/TEDA.C ^b^ 0.0005/4.7 wt%	Solution casting	13.8/(0.06) @ 1 MHz	Energy storage applications	[167]

^a^ Multi-walled carbon nanotube, ^b^ TEDA.C: Ferroelectric [Hdabco]ClO_4_.

**Table 5:** Comparison of properties between MXenes, graphene, and TMDCs.

Properties	MXenes	Graphene	TMDCs	Ref.
Conductivity	24,000 S/cm of Ti_3_C_2_T_x_	106 S/cm of pristine graphene	5.0 S/cm of MoS_2_	[49,60,178,179,180]
Surface functionality	Hydrophilic termination groups	Lack of surface termination groups	Lack of surface termination groups and π electrons	[181,182,183]
Young’s modulus (GPa)	483.5	1000	270	[169,184,185]
EMI shielding (dB)	92	35	—	[72,73]
Dispersibility	Stable water dispersibility	Easy to agglomerates	Easy to agglomerates	[186,187,188]

**Table 6:** A summary of studies on multifunctional MXene–polymer nanocomposites for electrode materials in supercapacitors.

Polymer Matrix	Nanofiller	Processing Method	Results	Refs.
PPy	Ti_3_C_2_T_x_	In situ polymerization	‑ Enhanced volume capacitance of 1000 F cm^−3^ and capacitance retention of 92% over 25,000 cycles.	[238]
PEDOT: PSS	Mo_1.33_C MXene	Self-assembly	‑ Flexibility, super high electrical conductivity; ‑ High volumetric capacitance of 568 F cm^−3^ at 0.5 A g^−1^; ‑ Simultaneous improvements in the energy density of 33.2 mWh cm^−3^ and power density of 19,470 mW cm^−3^.	[239]
i-PANI	Ti_3_C_2_T_x_	In situ non-oxidative polymerization	‑ Rapid and effective ion transfer; ‑ Flexibility and superior mechanical durability; ‑ High capacitance retention rate of 99.17%; ‑ Simultaneous enhancements in the volumetric capacitance, energy density, and power density.	[240]
PANI	graphene encapsulated Ti_2_CT_x_	In situ polymerization	‑ High specific capacitance of ~1143 F cm^−3^ at 1 A g^−1^; ‑ Capacitance retention of 97.54% after 10,000 cycles; ‑ Enhanced cycle stability of 94.25% after 10,000 cycles at 10 A g^−1^; ‑ Improved energy density and power density at 42.3 Wh kg^−1^ and 950 W kg^−1^, respectively.	[22]
PANI	3D macroporous Ti_3_C_2_T_x_	In situ polymerization	‑ Achieving ultra-high-rate capability and excellent volume capacitance of 1632 F cm^−3^ at 10 mV s^−1^.	[241]
PANI	Ti_3_C_2_T_x_	Scraper coating technology	‑ High volume capacitance of 1167 F cm^−3^ at 5 mV s^−1^; ‑ Extending the operating voltage window to 0.8 V; ‑ High energy density of 65.6 Wh L^−1^ at a power density of 1687.3 W L^−1^.	[242]
PEDOT: PSS	Ti_3_C_2_T_x_	Solution mixing	‑ Improved mechanical properties while maintaining flexibility; ‑ Achieving a high energy density of 23 mWh cm^−3^ at a power density of 7659 mW cm^−3^.	[243]

**Table 7:** A summary of studies on multifunctional MXene–polymer nanocomposites for capacitors.

Polymer Matrix	Nanofiller	Content	Results	Refs.
PVDF	BT–MXene	8 wt%/ 2 wt%	‑ Dielectric constant of 77, dielectric loss of 0.15 at 100 Hz; ‑ Breakdown strength of 220 MW m^−1^	[254]
PVC	MXene	10 wt%	‑ Dielectric constant of 11,800 and tan δ of 1.31 at 25 Hz; ‑ Thermal conductivity of 3.84 W mK^−1^; ‑ Improved mechanical performance and thermal stability.	[255]
Epoxy	MXene	40 wt%	‑ Low tan δ of 0.02; ‑ In-plane thermal conductivity of 1.29 W m^−1^ K^−1^ (10.65 times increase compared to pure epoxy).	[256]
PVA	MXene (V_2_C)	4 wt%	‑ Dielectric constant of ~24 and tan δ of ~0.14 (16 and 1.5 times higher than pure PVA, respectively), breakdown strength of ~31 (65% of pure PVA); ‑ Environmentally friendly.	[257]
PVA	MXene–CNNR	3.5 wt%	‑ High discharge energy density of 18.11 J cm^−3^, dielectric constant of 25.1, and tan δ of 0.03 at 1000 Hz; ‑ Improved tensile strength, Young’s modulus, and thermal conductivity.	[258]
PVDF Multilayer film	Ti_3_C_2_T_x_	—	‑ Dielectric constant of 41, tan δ of 0.028 at 1 kHz, and breakdown strength 284 MW m^−1^.	[259]
PMMA	MXene–ZnO	4 wt%/ 2 wt%	‑ Dielectric constant of 437 and tan δ of 0.36 at 25 Hz; ‑ Improved thermal stability and 14 times increase in thermal conductivity	[260]
PVDF	MXene@ MoS_2_	3.47 wt%	‑ Dielectric constant of 24.3 and dielectric loss (tan δ) of 0.02 at 103 Hz; ‑ High breakdown strength of 424.11 MW/m and energy density of 17.22 J cm^−3^, around 4.5 times higher than pure polymer.	[261]
P(VDF-HFP)	MXene/TiO_2_/MoS_2_	8 wt%	‑ High dielectric constant of 944 and low dielectric loss of 0.19; ‑ Enhanced mechanical properties and thermal stability.	[262]
PVC	V_2_C/Cu_2_O	7 wt%	‑ Improved dielectric constant of ~55 and low dielectric loss ~0.085 at 100 Hz; ‑ High breakdown strength of ~332 MV m^−1^.	[263]
PI	Oxidized MXene	0.5 wt%	‑ Significant energy density of 5.46 J cm^−3^ at high temperature of 100 °C and 2.05 J cm^−3^ at 150 °C compared to pure PI (1.28 J cm^−3^ at 100 °C).	[264]
PI	MXene@CTAB	7 wt%	‑ Enhanced dielectric constant of 7.8 and very low dielectric loss of 0.027 at 100 Hz; ‑ Significant thermal stability, low water absorption, and good hydrophobicity.	[265]
PVC	Ti_2_C–diamond	12 wt%/4 wt%	‑ Increase in dielectric constant (~153), low dielectric loss (~0.14), and high breakdown strength (~312 MV m^−1^) at 100 Hz.	[266]
Acrylic resin elastomer	acidified CNT@ MXene	1.42 vol%	‑ High dielectric constant of 120 and low tan δ of 0.15 at 100 Hz; ‑ Good mechanical properties.	[267]
SR	MXene decorated with Ag	3.2 g	‑ High dielectric constant of 7.29, low tan δ of 0.00114 at 1000 Hz; ‑ Remarkable mechanical properties, tensile stress 554 kPa and elongation at break of 257%.	[268]

**Table 8:** A summary of studies on fabric-based multifunctional MXene materials for sensing and Joule heating applications.

Substrate Fabric/Medium	Filler(s)	Main Functionality	Results	Refs.
Nylon and PU nanoyarns	Ti_3_C_2_T_x_ MXene	Piezoresistive motion sensing	‑ GF of 17 for a tensile strain range of 20–50% in MXene–PU nanoyarn containing 19 wt% MXene; ‑ Stretchability of 43% and 263%, for MXene–Nylon and MXene–PU nanoyarns, respectively; ‑ Electrical conductivity of 1195 S/cm and 79 S/cm for MXene–Nylon and MXene–PU nanoyarns, respectively.	[278]
Cellulose-based yarns (cotton, bamboo, linen)	Ti_3_C_2_T_x_ MXene	Capacitive pressure sensing	‑ Specific capacitance of 759.5 mF/cm at 2 mV/s, GF of 6.02, and stable performance for over 2000 cycles; ‑ High electrical conductivity of 440 S/cm with 77 wt% MXene loading; ‑ Proven knittability using an industrial knitting machine with the interlock knitting method; ‑ Proven washability of the coated yarns by washing for 45 cycles at 40 °C to 65 °C, with a minimal increase in electrical resistance.	[279]
Cellulose nonwoven fabric	Ti_3_C_2_T_x_ MXene	piezoresistive pressure sensing	‑ Sensitivity of 28.72 kPa^−1^ within sensing range of 0–17.4 kPa, with durability exceeding 2000 cycles; ‑ Fast response time of 0.5 s and recovery time of 20 ms for real-time human activity monitoring, like finger pressing, walking, and beating pulse; ‑ EMI-shielding effectiveness of the sample coated and padded nine times was recorded at 35.2 dBl ‑ Electroactive properties: the highest temperature recorded was 146.7 °C at 5 V, making it suitable for personal heating devices.	[280]
Carboxymethyl chitosan-coated cotton fabric	Ti_3_C_2_T_x_ MXene	fire-warning/Piezoresistive motion sensing	‑ Fire alarm was activated using the thermoelectric properties of MXene, where a temperature difference results in a voltage variation, triggering the alarm rapidly within 3.8 s without the need for an external power source; ‑ Piezoresistive motion sensing for different motion modes, implying stable resistance changes under varying strain (1.5–4.5%), well distinguishing between different motions; ‑ Flame retardancy by synergistic carbonization of MXene and CCS, reducing the peak heat release rate and LOI by 66.9% and 45.5%, respectively; ‑ Joule heating of the fabric showed a surface temperature of ~75 °C at 4.5 V.	[281]
PET-based textile	PPy-modified MXene	Piezoresistive motion sensing	‑ Strain sensing for two modes: torsion (from 0° to 540°) and bending (with chord lengths ranging from 4 cm to 2 cm); ‑ Electrical conductivity of 1000 S/m with 13 wt% nanomaterial loading; ‑ EMI shielding for one piece of textile coated ten times was 42 dB, with a big jump to 90 dB for three pieces; ‑ Electrothermal performance at 4 V resulted in a surface temperature of 79 °C; ‑ Silicone coating increased contact angle from 57° to 126°, ensuring long-term use in high-humidity conditions.	[282]
PDA@ polypropylene textile	Ti_3_C_2_T_x_ MXene	Strain/temperature sensing	‑ Strain-sensing capability for detecting human motion monitoring in different modes of elbow bending, fisting, walking, and running with a GF of 18, suitable for strains up to 45% and durable for over 500 cycles; ‑ Temperature sensing performance with a TCR of −1.8%/°C, operating between 25 °C to 100 °C; ‑ Electrical conductivity of 120 S/m for 8-times-coated textiles. ‑ Joule heating behavior: surface temperature of 89.4 °C at 14 V; ‑ Super hydrophobicity: contact angle of 151.4°; ‑ Breathability: vapor transmission rate of 0.49 kg/m^2^ h	[283]
PDAC-treated cotton	MXene–Ag NWs	Pressure sensing	‑ Pressure sensing: GF of 2.32 kPa^−1^ across a wide range of 2 to 120 kPa with a durable performance over 2000 cycles; ‑ Different human movements detection, such as puffing, wrist bending, clicking, finger bending, neck bending, heart pulse, and vocal signals like “good morning” and “SOS”; ‑ EMI shielding of the 15-times-coated fabric showed an effectiveness of 40 dB, dominated by absorption; ‑ Joule heating performance at 4.5 V reached 63 °C with stable performance over multiple cycles.	[284]
Cotton	MXene	Pressure sensing	‑ Pressure sensitivity of the decorated fabric with 2 wt% of MXene was 1.16 and 3.18 for low and high strain rates, respectively, enabling the detection of various motions and vocal signals of different letters with clear distinguishability; ‑ Electrothermal performance of the coated fabric with 6 wt% MXene: Surface temperature of 29 °C at 1 V and 150 °C at 6 V; ‑ EMI-shielding effectiveness of the decorated fabric with 6 wt% MXene was recorded at 36 dB across the X-band.	[285]
Cotton MXene/silicon nanocomposite	MXene–TiO_2_–MoS_2_	TENG self-powered wearable electronics	‑ The sensing capability of the fabricated micro-patterned fabric was based on voltage generation under different force modes; ‑ TENG specification: 1.47 kV at 6 N and 4 Hz; Current and charge density at an MXene concentration of 3 mg/cm^2^: 200 mA/m^2^ and 980 mC/m^2^, respectively.	[286]
Cellulose nonwoven fabric	CNT–MXene	Pressure sensing	‑ GF of 0.245 kPa^−1^ for pressures within a range of 0.128–1.9 kPa, with well-distinguishability between different objects with different weights. ‑ EMI-shielding effectiveness: 30 dB across the X-band, dominated by absorption (63.3%) for 9-cycle-coated sample; ‑ Thermoelectric performance: a surface temperature of 70.9 °C at 5 V.	[287]
Cotton	MXene	Pressure sensing/wearability	‑ GF of 12.095 kPa^−1^ and 3.844 kPa^−1^ for pressures between 29–40 kPa and below 29 kPa, respectively, with stable performance for 5600 cycles; ‑ Response time of 26 ms and recovery time of 50 ms.	[288]
Cotton	RGO–Ti_3_C_2_T_x_ MXene	Piezoresistive strain sensing	‑ Strain-sensing performance: Negative GF of −7.67 with well-distinguishability between different signals like bending of different joints; The most relative resistance change was recorded for finger joint bending up to −85.6%. ‑ Energy storage performance: gravimetric specific capacitance of 683.3 F/g and areal specific capacitance of 298 mF cm^−2^ for the fabric coated in 3 cycles; ‑ EMI-shielding effectiveness of 29.04 dB across the X-band, dominated by absorption; ‑ electrothermal performance: surface temperature of 66.7 °C at 12 V; heating efficiency: DT = 36 °C for 4 cycles of MXene spray coating.	[289]
Silk textile	Ti_3_C_2_T_x_/Ag NWs	Humidity sensing	‑ Humidity sensing performance: response time of 5 s and recovery time of 80 s at 57% room humidity; ‑ EMI-shielding effectiveness: 42 dB for a single layer (120 mm) and 90 dB for 4-layered samples, both with an Ag NWs concentration of 1 mg/mL and 10 coating cycles.	[290]

**Table 9:** A summary of studies on hydrogel-based multifunctional MXene materials for sensing and Joule heating applications.

Matrix Hydrogel	Filler(s)	Main Functionality	Results	Refs.
PVA	Ti_3_C_2_T_x_ MXene	Piezoresistive and capacitive sensing for E-skins	‑ Capacitive strain sensor: GF of 0.4, with great linearity up to 200% strain and only a 5.8% reduction in capacitance retention over 10,000 cycles, capable of detecting delicate movements with low hysteresis, like eye blinking and pulse detection; ‑ Piezoresistive sensor showed considerable hysteresis; ‑ Stretchability of 43% and 263% for MXene–Nylon and MXene–PU nanoyarns, respectively; ‑ Stretchability: strain at break of 1200%; ‑ Self-healing: super quick self-healing of 0.15 s.	[291]
PAAM/PVA organo-hydrogel	Ti_3_C_2_T_x_ MXene	Strain sensing	‑ Strain sensing: GF of 5.02 and 44.85 for strain ranges of 0–200% and 200–350%, respectively. The strain detection limit was 0.1%, enabling the sensor to detect subtle movements, like biological signals; ‑ Anti-freezing property: maintaining its stretchability at –40 °C by incorporating polyethylene glycol; ‑ Self-healing property: retaining 85% of tensile strength in 12 h, while electrical resistance was fully restored within 3.1 s.	[292]
PAA	Ti_3_C_2_T_x_ MXene	piezoresistive pressure sensing	‑ GF of 0 to 5 as strain increased with high sensitivity starting from 1% strain. Different movement detection such as finger and elbow bending, swallowing, forehead movements, water droplet falling, and speech recognition; ‑ High electrical conductivity of 0.8 S/m for 12.2 wt%; ‑ EMI-shielding effectiveness: 45.3 dB for the sample with 8.5 wt% MXene (0.13 mm thickness) across terahertz frequency; ‑ Excellent stretchability and rapid self-healing within seconds, with tensile toughness and electrical resistance fully restored within 10 min.	[293]
PAAM organo-hydrogel	Ti_3_C_2_T_x_ MXene	strain sensing	‑ Strain-sensing performance: GF of 6.31 for large strains with high sensitivity and stable relative resistance change over 500 cycles; ‑ Anti-freezing property: operating in subzero conditions (−20 °C without cracking or losing flexibility).	[294]
Polyampholytes	Ti_3_C_2_T_x_ MXene	Wearable epidermal sensor	‑ Strain sensing: GF of 2.34 and 6.31 for strain ranges of 0–200% and 200–1000%, respectively. Detection of different human body activities, like different joint bending, blinking, swallowing, and speech cognition; ‑ Self-adhesion to various substrates, with adhesion strength decreasing from approximately 13 to 7 kPa in the order of glass > iron > PET > porcine skin; ‑ Excellent self-healing and post-healing stretchability, and electrical conductivity.	[56]
PAAM/PVA double network hydrogel	Ti_3_C_2_T_x_ MXene	Pressure/strain sensing	‑ Strain-sensing performance: GF of 1 and 0.6 for 0–250% and 300–450% strain ranges. Good distinguishability for various types of movements, like gestures, facial expressions, and different vocal fold vibrations; ‑ Pressure sensing: The fabricated E-skin could estimate the extent of pressure, map the pressure distribution, and distinguish between the number of tapped areas; ‑ TENG performance: an open-circuit voltage of 180 V, a short-circuit current of 10 μA, and a transferred charge of 65 nC; ‑ Eight-times-coated textile showed electrical conductivity of 120 S/m; ‑ Joule heating behavior: surface temperature of 89.4 °C at 14 V; ‑ Super hydrophobicity: contact angle of 151.4°; ‑ Breathability: vapor transmission rate of 0.49 kg/m^2^ h.	[295]
